# Assessing the acceptability of village health workers’ roles in improving maternal health care in Gombe State, Nigeria a qualitative exploration from women beneficiaries

**DOI:** 10.1371/journal.pone.0240798

**Published:** 2020-10-22

**Authors:** Maryam Al-Mujtaba, Olukolade Shobo, Bolanle C. Oyebola, Benson O. Ohemu, Isaac Omale, Abdulrahman Shuaibu, Jennifer Anyanti

**Affiliations:** 1 International Research Center of Excellence, Institute of Human Virology Nigeria, Abuja, Federal Capital Territory, Nigeria; 2 Monitoring and Evaluation Department, Society for Family Health, Abuja, Federal Capital Territory Nigeria; 3 Programmes Department, Society for Family Health, Abuja, Federal Capital Territory, Nigeria; 4 Communications Department, Society for Family Health, Abuja, Federal Capital Territory, Nigeria; 5 Office of the Executive Secretary, Gombe State Primary Healthcare Development Agency, Gombe, Gombe State, Nigeria; 6 Office of the Deputy Managing Director, Society for Family Health, Abuja, Nigeria; Kwame Nkrumah University of Science and Technology, GHANA

## Abstract

**Introduction:**

Maternal, and under-five mortality rates in Gombe State are disproportionately high. The Society for Family Health (a Non-Governmental Organization) in collaboration with Gombe State Primary Health Care Development Agency implemented the Village Health Worker (VHW) Program in Gombe to address the low uptakes of maternal neonatal and child health (MNCH) services and reduced the impact of healthcare worker insufficiency. VHWs are lay indigenous women trained to educate and encourage women to use MNCH services, provide simple community-based maternal and new-born care through home visits, and facilitate facility linkage. We assessed the acceptability of VHW services among women beneficiaries of the Program.

**Methods:**

Qualitative data were obtained through six focus group discussions with 58 women beneficiaries of the VHW program who delivered within the last 12 months preceding study period (October–November 2018). Themes explored were roles and acceptability of VHWs, and the influence of VHWs on the uptake of MNCH services. We analyzed data with NVivo 12, using Grounded Theory.

**Results:**

Participants’ mean age was 25.1 (± 5.3) years old. Most participants 39 (67%), had been in contact with a VHW for at least 10 months. VHWs visited pregnant women at home and registered them for antenatal care, provided them basic maternal healthcare, health education, and facilitated facility linkage. Participants generally accepted the VHW Program because it was community-based, VHWs were indigenous community members, delivered clear messages, and influenced husbands and mothers-in-law to support women’s’ use of MNCH services. VHWs’ interventions were perceived to have improved health literacy and the uptake of MNCH services. Participants generally admired the VHW occupation and recommended VHW program scale-up, and for VHWs to be offered basic obstetric training and employment by health facilities or the government.

**Conclusion:**

The general acceptance and positive views of VHWs from beneficiaries of the program demonstrates the feasibility of the program to improve the uptake of MNCH services.

## Introduction

The use of skilled birth attendants and access to emergency obstetric care which are vital for reducing maternal and newborn deaths [1], hinge on the availability of skilled health workers and effective Human Resources for Health (HRH) Management [2]. In the last two decades, accumulating evidence support the vital role that Community Health Workers (CHWs) play to improve Maternal Neonatal Child Health (MNCH), and bridge gaps in HRH shortages [3–5]. CHWs are a diverse category of low-cadre healthworkers [4], who receive standard training on specific topics of shorter duration than health professionals, are familiar with local indigenous context, and work within the community at household levels to promote healthy behaviors, provide basic services and to facilitate linkage to the facility [5–13]. Countries that made significant progress towards MDGs 4 and 5 had strong national CHW programs [4]. CHWs are equivalent to Village Health Workers (VHWs) in Nigeria.

Nigeria, in sub-Saharan Africa, has the highest (67 000) and second highest (100 per 1,000 live births) maternal [14], and under five deaths globally [15]. The country has a population of over 190 million (2018 estimate) [16], and over 7 million births occurring annually [17]. However, there is dearth in the quality, quantity and mix of health care workers with a skewed distribution towards urban and southern populations [18]. Gombe State, in North-East Nigeria has maternal and under five mortality rates of 1002 per 100,000 live births and 104 per 1000 live births respectively [19]. These are higher than the national maternal and under five mortality rates of 917 per 100 000 live births [14], and 100 per 1000 live births respectively [15]. Furthermore, the State’s total health care worker density of about 1 per 1,000 [19], is lower than the national health worker density of 2.52 per 1,000 [20], and much lower than the WHO standard of 4.45 per 1000 population [21].

### VHW program implementation

**Fig 1:**
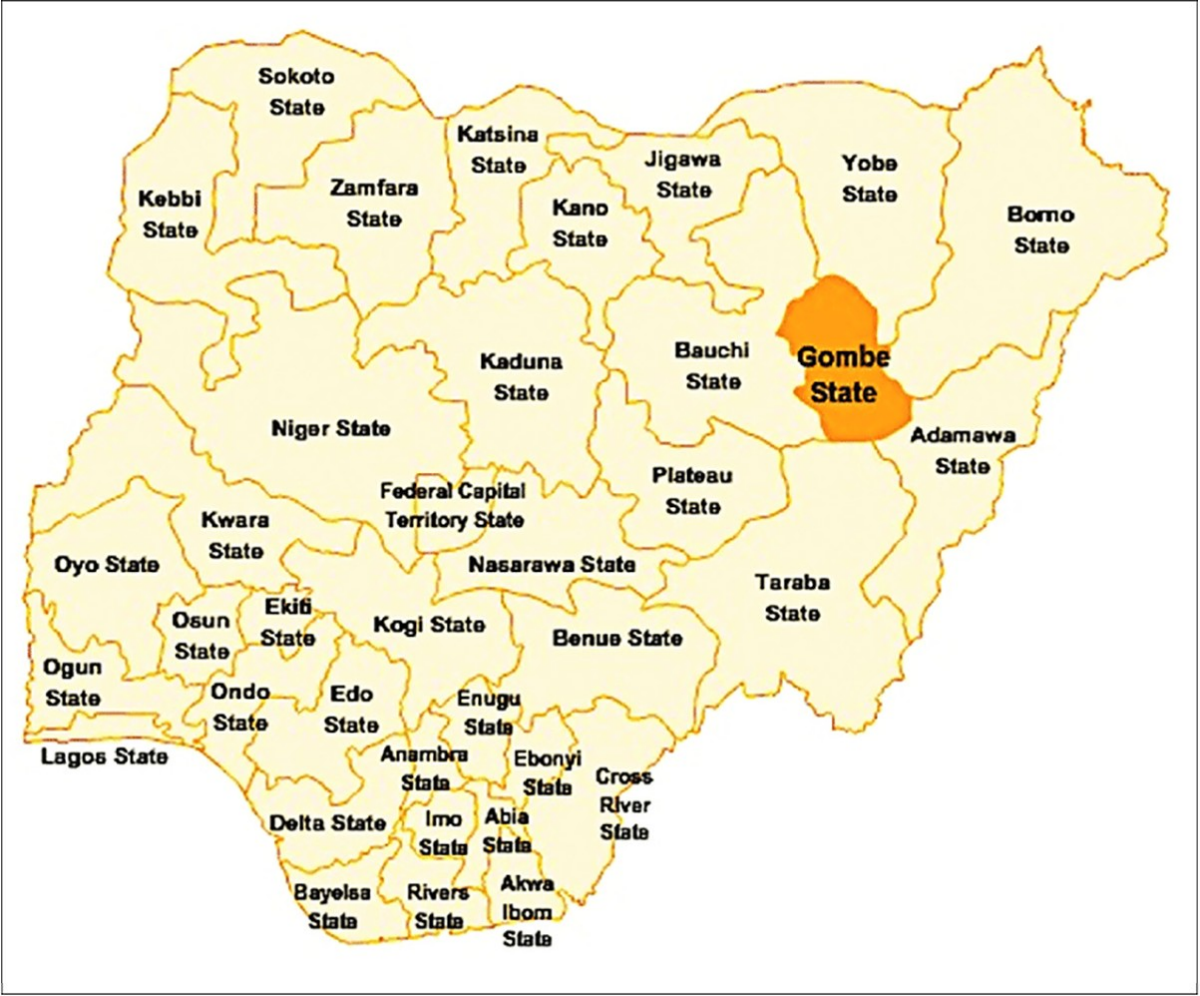
Map of Nigeria showing Gombe State.

**Fig 2:**
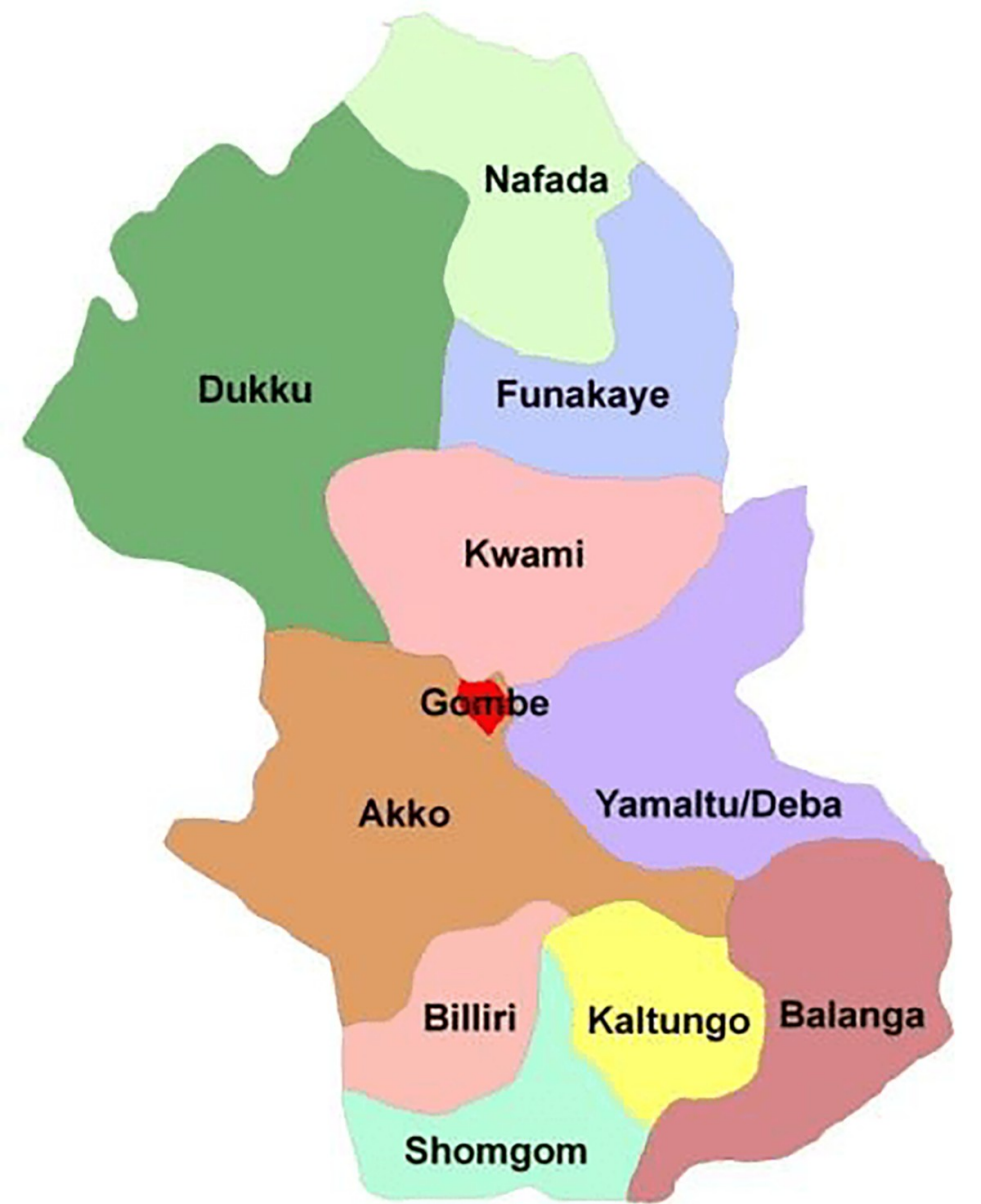
Map of Gombe State showing the 11 Local Government Areas.

Gombe State, in North-East Nigeria (Fig 1) has 565 health facilities across 11 Local Government Areas (equivalent to a district) (Fig 2). Each Local Government Area contains 10–11 political wards (114 wards) [22]. The ward is the operational unit for implementing primary healthcare activities because it typically has a population of 10,000 to 30,000 people [23], is politically homogenous, and has well-defined boundary making it easier to facilitate political and community support instead of the district. The Ward Development Committee constitutes of selected community residents, and serves as the ward’s supreme body for the functions of the health centre and other development activities [24].

In October 2016, the Society for Family Health a Nigerian Non-Governmental Organization in collaboration with the Nigerian government through the Gombe State Primary Health Care Development Agency (the Agency), implemented the VHW Program among 57 of the114 wards of Gombe State. The 57 selected wards were identified as ‘priority’ by the Agency because of their weak maternal health indicators [24, 25].

Women selected to be trained as VHWs will have met the following criteria:

1. Age- 15yrs and above (the selection criteria included minors (15-year olds) because in Gombe State, many 15-year olds are married mothers, and socially considered as adults)
2. Fluent in the local language (Hausa)
3. Minimal educational qualification: could read and write in English or Hausa
4. Preferably married and with permission from husband (to avoid domestic discordance/conflict)
5. Resided in the community
6. Were familiar with norms and values of the community
7. Agreed to link activities to ward facilities

Based on the above-mentioned criteria, the Ward Development Committees with guidance from the Agency and Local Government Areas selected 1200 women to be trained as VHWs. Selected women were exposed to a three-week (classroom and field) intensive training. The training was implemented in two phases to allow for gradual learning of low literate cadre of community volunteers. The first phase focused on maternal and newborn health while the second phase incorporated the child health component in addition to communicable and non-communicable diseases. There was a six-month interval between the first and second training phases. Training was guided by the VHW training curricula (Table 1).

**Table 1:** VHW training curricula.

Module	Module Topic	Sessions
**PHASE– 1 OF THE PROGRAM**		
Module A	Introduction and Community Outreach/Mobilization	Session 1: Training goal and objectives
		Session 2: Community Outreach/Mobilization
Module B	Maternal and Newborn Health	Session 3: Antenatal Care
		Session 4: Labour and Delivery
		Session 5: Post Natal Care
		Session 6: Common Newborn Problems
		Session 7: Healthy Timing and Spacing of Pregnancy
Module C	Nutrition	Session 8: Maternal Nutrition
		Session 9: Infant and Young Child Feeding (IYCF)
		Session10: Micro nutrition supplementation for mother and baby
		Session11: Assessment and classification of children with malnutrition
Module D	Water, Sanitation and Hygiene	Session 12: Adequate and Clean water
		Session 13: Sanitation
		Session 14: Hygiene
**PHASE– 2 OF THE PROGRAM**		
Module E	Child Health	Session 15: Immunization
		Session 16: Pneumonia
		Session 17: Diarrhoea
		Session 18: Fever/Malaria
Module F	Communicable and Non-Communicable Diseases	Session: 19: Cholera
		Session 20: Tuberculosis
		Session 21: HIV
		Session 22: Diabetes Mellitus
		Session 23: Hypertension
		Session 24: Malaria
Module G	First Aid	Session 25: Introduction to First Aid
		Session 26: Wound Management
Module H	Referral	Session 27: Introduction to Referrals
Module I	Record Keeping	Session 28: Record keeping

After the training, VHWs were branded, provided teaching resources, and deployed to their various communities to enhance MNCH services in the communities and at household levels. VHWs were required to interact with a pregnant woman at least four times during pregnancy, twice post-delivery and to provide basic household level MNCH services and linkage to facility. VHW-client interactions were guided with pictorial flip charts (with captions written in Hausa language). Ideally, the VHW density was 2 per 1000 population but usually adjusted to the communities population and peculiarities [24]. The Agency financed each VHW’s monthly stipend of NGN 6000 ~ USD16.5 (at exchange rate ₦365 to USD1). VHWs were supervised and mentored by selected and trained Community Health Extension Workers employees of health facilities within the VHW wards. Community Health Extension Workers are trained in Schools of Health Technology for either a 2- or 3-year training program, the latter featuring more training in the actual delivery of infants [26]. One Community Health Extension Worker supervised 10 VHWs. Furthermore, supervision and performance tracking of VHWs was conducted by Society for Family Health through 11 Program Officers (one Program Officer per Local Government Area), and the Ward Development Committees tracked VHWs performance in their wards and provided feedback to their communities and to the Local Government Areas’ Maternal Child Health Coordinators [24].

Earlier studies have shown that VHWs were usually accepted when the community considered them approachable, competent, trustworthy, and if VHWs provided community-based and culturally appropriate services [13, 27–30]. In the same vein, VHWs were found unacceptable when they came from out-side communities [27], or were perceived as incompetent or not trustworthy [28–30]. Since community acceptability of VHW roles is central to its success [13, 27–30], the aim of this study was to assess the acceptability of the VHW’s roles among women beneficiaries of the program in Gombe State.

## Methods

### Study design

This was a cross-sectional qualitative study conducted employing focus group discussions among women of reproductive age beneficiaries of the VHW program residing in rural areas of Gombe State.

### Study setting

Gombe State covers an area of 20,265sq, km [31], population is over 3.2 million (2016 estimate) [17], with approximately 136,000 births yearly [32], the State’s fertility rate (7.3) is higher than the national fertility rate (5.8) [33]. Uptake of facility antenatal (44.4%) and delivery (27.7%) [34], are lower than the national averages of 49.1% [35] and 39% respectively [34]. Hausa language is the inter-ethnic medium of communication, and the three common occupations are farming, cattle-herdsmen-ship and trading. Most residents (72.2%) live under 1USD/day [31], and literacy rates among males and females are 47.5% and 37.5% respectively [33].

### Population, selection of wards and eligibility criteria

The study population consisted of women residents of Gombe State who have benefited from the VHW Program. Society for Family Health monthly monitoring data on facility delivery uptake among VHW beneficiaries guided the selection of study VHW wards. After 23 months (October 2016 –September 2018) of VHW implementation, mean uptake of facility delivery services among VHW beneficiaries was 65% (±17.6%). The three wards that represented the maximum (Banganje North—96%), mean (Akko 65%) and minimum (Zange 23%) uptake of facility delivery services among the 57 VHW intervention wards, were selected for the study (Table 2).

**Table 2:** Selected VHW wards, number of VHWs and facility delivery uptake.

VHW Intervention Ward	Population	Number of VHWs	Facility Delivery Uptake
Bangaje North	27,171	23	96%
Akko	32,316	33	65%
Zange	25,038	26	23%

Women were eligible for the study if they had delivered either at home or in the facility 12 months prior the study period and were residents of the study selected wards: Baganje North, Akko, or Zange. (Table 2).

#### Recruitment

Participants were recruited by Society for Family Health’s Program Officers through the VHWs. For each study selected ward, the Program Officer responsible for that ward purposefully selected a VHW who identified focus group discussion (FGD) eligible clients and verbally invited them to participate in the focus group. Recruitment was stopped once a target of 10 women had been reached for each FGD. Interested participants showed up for the FGD on the appointed date and time. For each selected ward, the first FGD targeted women who delivered in the facility (facility group), and the second FGD targeted women who delivered at home (home group). However, in Baganje North with an almost 100% facility delivery uptake, both FGDs consisted of women who delivered in the facility. Furthermore, the first Baganje North Group had only seven participants. Three participants were away at farm harvesting activities. While the second Baganje North Group had 11 participants because one of the three participants unable to join the first group joined the second group. In aggregate, there were a total of 58 participants, four facility groups (two from Baganje North, one from Akko and one from Zange) and two home groups (one from Akko and one from Zange).

#### Research team

Seven bilingual (English and Hausa speakers) researchers were involved in data collection: author MAM (female, MPH, and over one year conducting and analysing qualitative data in a similar study setting and over 5 years research experience) was the Research Consultant responsible for the overall design and conduct of the study. Six Research Assistants who work as independent consultants: five female and one male were university graduates with a minimum of one year working experience facilitating FGDs on a number past VHW program evaluations which generally involved data collection from VHWs. The six Research Assistants also received one-day training facilitated by MAM in qualitative data collection for the purpose of the study and were financially remunerated for three days they were contracted for data collection. None of the researchers had prior contact or any kind of relationship with the FGD participants.

#### Data collection tools

A one-page 5-minute survey with open and close ended questions was designed to capture participants’ sociodemographic information (age, education, occupation), obstetric history numbers of facility and home deliveries and duration of contact with a VHW. For the FGD guide, multi-theme, semi-structed questions with probes were developed. Both data collection tools were updated after input were received through email from co-authors OS, BCO, BOO, IO and JA (Society for Family Health Staff directly involved with coordinating and monitoring the VHW program), to reflect their input of rephrasing the questions to accommodate the cultural context of study population. The first section of the FGD moderator guide explored topics on participants’ access, use, and satisfaction with facility delivery services. The second part of the guide examined roles of VHWs, and participants’ acceptability of the VHW program by asking questions on experiences/views on VHWs’ services, messages, communication skills, influence MNCH service use, and recommendations for the program. Hausa translated versions of both data collection tools were developed and those versions were used during data collection. This manuscript is reporting only data collected in the second half of the data collection tool that centered on roles and participant’s acceptability of VHW program.

### Ethics

Ethical approval was obtained from the Gombe State Ministry of Health and all participants provided written or verbally documented consent. Prior to each FGD, one of the Research Assistants or MAM eloquently explained the content of the study information sheet and consent form to all participants in Hausa. After thorough explanation of the study information, participants were required to sign the consent to document that they have agreed to participate in the Study. Participants who were non-literate, were required to give the Research Assistants verbal consent to initialize the consent form and tick the verbal consent checkbox on their behalf. Participants were assured of their anonymity and that their response will not affect their interactions and/or care given to them by the VHWs and facility healthcare workers. All FGDs were audio recoded and notes taken with the consent of the participants.

To maintain anonymity and establish a conducive atmosphere for discussion, participants used self-chosen aliases for each FGD.

#### Data collection

Researchers introduced themselves to study participants as independent consultants not affiliated with the VHW program or the health facility, and the reason for conducting the study was to improve VHW services. Prior to the commencement of FGDs, sociodemographic information of each participant was collected by administering the 5-minute survey in Hausa language. In total, six FGDs were conducted: two FGDs per ward. MAM and two Research Assistants conducted each of the FGDs. First Research Assistant and MAM moderated and co-moderated the discussion, while the second Research Assistant observed the discussion and noted participants’ non-verbal cues and synergistic group effects. Discussions were conducted either in open outdoor settings or private rooms within the premises of a selected health facility during non-working hours. Only researchers and participants were present during FGD discussions. All FGDs were conducted in Hausa, audio recoded, and notes taken with the consent of the participants. No repeat FGDs were conducted, and participants were not required to give feedback on findings. Duration of FGDs ranged from 40–90 minutes and participants were given refreshment worth NGN 500 ~ USD1.3 (at exchange rate ₦365 to USD1) at the end of the FGDs. The qualitative study time was 30^th^ October 2018 to 1^st^ November 2018. Daily debriefing sessions were held with data collectors to discuss findings and identify saturation of themes. The target was to conduct six FGDs with the plan to conduct more in the instance of non-saturation of data. However, conducting more FGDs was not required as data saturation was reached within the six FGDs.

#### Data storage and analysis

Audio-recorded FGD transcripts, signed informed consent forms and completed socio-demographic surveys were filed and locked in a secure cupboard and were only accessible to the research team. Socio-demographic data were analysed with Microsoft excel formula function: age mean, and age standard deviation were calculated. Other information was presented in aggregate and percentages (Table 3).

**Table 3:** Sociodemographic, obstetric history, place of delivery & duration of contact with VHWs.

	Group 1	Group 2	Group 3	Group 4	Group 5	Group 6	All Groups
**Sample Size**	N = 7	N = 11	N = 10	N = 10	N = 10	N = 10	N = 58
Age, years: mean (SD)	28.0 (± 4.0)	30.4 (± 4.2)	24.0 (± 3.0)	25.0 (± 5.0)	21.1 (±4.4)	23.0 (± 4.1)	25.1 (± 5.3)
**Other Characteristics: n (%)**						
**VHW Intervention Ward**	(BFDG1) ^a^	(BFDG2) ^b^	(AFG)^c^	(AHG)^d^	(ZFG)^e^	(ZHG)^f^	All selected wards
**Place of last delivery**							
Facility	7 (100.0)	11 (100.0)	9 (0.0)	0 (0.0)	10 (100.0)	0 (0.0)	37 (63.7)
Home	0 (0.0)	0 (0.0)	1 (10.0)	10 (100.0)	0 (0.0)	10 (100.0)	21 (36.2)
**Formal Education**							
None	1 (14.3)	1 (9.0)	1 (10.0)	3 (30.0)	4 (40.0)	0 (0.0)	10 (17.2)
Informal Schooling^g^	0 (0.0)	0 (0.0)	5 (50.0)	0 (0.0)	0 (0.0)	9 (90.0)	14 (24.1)
Primary School	0 (0.0)	3 (27.0)	2 (20.0)	4 (40.0)	4 (40.0)	1 (10.0)	14 (24.1)
Secondary School	6 (85.0)	7 (64.0)	2 (20.0)	3 (30.0)	2 (20.0)	0 (0.0)	20 (34.4)
**Occupation**							
None	1 (14.3)	5 (45.0)	3 (30.0)	8 (80.0)	9 (90.0)	4 (40. 0)	30 (51.7)
Business/Trade	1 (14.3)	0 (0.0)	7 (70.0)	2 (20.0)	0 (0.0)	6 (60.0)	16 (27.5)
Professional^h^	0 (0.0)	0 (0.0)	0 (0.0)	0 (0.0)	1 (10.0)	0 (0.0)	1 (1.7)
Farmer	5 (71.0)	6 (55.0)	0 (0.0)	0 (0.0)	0 (0.0)	0 (0.0)	11 (18.9)
**Religious Affiliation**							
Christianity	7 (100.0)	11 (100.0)	0 (0.0)	0 (0.0)	0 (0.0)	0 (0.0)	18 (31.0)
Islam	0 (0.0)	0 (0.0)	10 (100.0)	10 (100.0)	10 (100.0)	10 (100.0)	40 (69.0)
**Ethnicity**							
Fulani	0 (0.0)	0 (0.0)	8 (80.0)	9 (90.0)	2 (20.0)	2 (20.0)	21(36.2)
Tangale	7 (100.0)	11 (100.0)	0 (0.0)	0 (0.0)	0 (0.0)	0 (0.0)	18 (31.0)
Others^i^	0 (0.0)	0 (0.0)	2 (20.0)	1 (10.0)	8 (80.0)	8 (80.0)	19 (32.7)
**Number of living children**							
None	0 (0.0)	0 (0.0)	0 (0.0)	0 (0.0)	0 (0.0)	0 (0.0)	0 (0.0)
1–2	2 (28.0)	3 (27.0)	2 (20.0)	5 (50.0)	5 (50.0)	4 (40.0)	21 (36.2)
3–4	4 (57.0)	4 (36.0)	5 (50.0)	3 (30.0)	4 (40.0)	3 (30.0)	23 (39.6)
5+	1 (14.3)	4 (36.0)	3 (40.0)	2 (20.0)	1 (10.3)	3 (30.0)	14 (24.1)
**History of facility delivery**							
None	0 (0.0)	0 (0.0)	0 (0.0)	2 (20.0)	0 (0.0)	9 (0.0)	11 (18.9)
1–2	5 (71.0)	2 (18.0)	2 (20.0)	6 (60.0)	7 (70.0)	1 (10.0)	23 (39.6)
3–4	2 (28.5)	5 (45.0)	5 (50.0)	1 (10.0)	3 (0.0)	0 (0.0)	16 (27.5)
5+	0 (0.0)	4 (36.0)	3 (30.0)	1 (10.0)	0 (0.0)	0 (0.0)	8 (13.7)
**History of home delivery**							
None	3 (43.0)	11 (100.0)	7 (70.0)	0 (0.0)	6 (60.0)	0 (0.0)	27 (46.5)
1,2	0 (0.0)	0 (0.0)	3 (30.0)	8 (80.0)	2 (20.0)	3 (30.0)	16 (27.5)
3+	4 (57.0)	0 (0.0)	0 (0.0)	2 (20.0)	2 (20.0)	7 (70.0)	15 (25.8)
**Use of Traditional Birth Attendant**							
None	4 (57.0)	11 (100.0)	9 (90.0)	2 (20.0)	6 (60.0)	0 (0.0)	32 (55.1)
1,2	3 (43.0)	0 (0.0)	1 (10.0)	7 (70.0)	2 (20.0)	3 (30.0)	16 (27.5)
3+	0 (0.0)	0 (0.0)	0 (0.0)	1 (10.0)	2 (20.0)	7 (70.0)	10 (17.2)
**Last delivery**							
Less than a month ago	0 (0.0)	1 (9.0)	2 (10.0)	2 (20.0)	0 (0.0)	0 (0.0)	5 (8.6)
1–3 months ago	3 (43.0)	3 (27.0)	2 (20.0)	6 (60.0)	3 (30.0)	1 (10.0)	18 (31.0)
4–6 months ago	2 (28.5)	1 (9.0)	2 (20.0)	1 (10.0)	4 (40.0)	5 (50.0)	15 (25.8)
7–9 months ago	0 (0.0)	3 (27.0)	3 (30.0)	1 (10.0)	2 (20.0)	2 (20.0)	11 (18.9)
10+ months ago	2 (28.5)	3 (27.0)	1 (10.0)	0 (0.0)	1 (10.0)	2 (20.0)	9 (15.5)
No response	0 (0.0)	0 (0.0)	1 (10.0)	0 (0.0)	0 (0.0)	0 (0.0)	1 (1.7)
**First Contact with VHW**							
Less than a month ago	0 (0.0)	0 (0.0)	0 (0.0)	0 (0.0)	0 (0.0)	0 (0.0)	0 (0.0)
1–3 months ago	1 (14.2)	1 (9.0)	0 (0.0)	0 (0.0)	0 (0.0)	0 (0.0)	2 (3.4)
4–6 months ago	1 (14.2)	0 (0.0)	2 (20.0)	3 (30.0)	0 (0.0)	0 (0.0)	6 (10.3)
7–9 months ago	2 (28.5)	1 (9.0)	2 (20.0)	4 (40.0)	1 (10.0)	0 (0.0)	10 (17.2)
10+ months ago	3 (43.0)	9 (82.0)	5 (50.0)	3 (30.0)	9 (90.0)	10 (100.0)	39 (67.2)
No response	0 (0.0)	0 (0.0)	1 (10.0)	0 (0.0)	0 (0.0)	0 (0.0)	1 (1.7)

SD–Standard Deviation

^a^Baganje North Facility Group 1

^b^Baganje NorthFacility Group 2

^c^Akko Facility Group 3

^d^Akko Home Group 4

^e^Zange Facility Group 5

^f^Zange Home Group 6

^g^Islamic or Bible School

^h^one TBA

^i^Include four Boboriya, three Hausa, five Karekare, three Bolewa, two Kanuri one Waja and one Tera

All six focus groups were conducted in Hausa. The six Research Assistants who conducted and observed the FGDs translated and manually transcribed the FDGs into English. For quality control, after the transcription of the first 2 FGDs, MAM reviewed the transcripts against the respective audio recordings to verify the quality of the translation and transcription. As transcriptions were considered satisfactory, the transcription process was continued. All the six transcripts were coded by the MAM using NVivo 12 (Pro for Windows), using the principles constant comparative method in grounded theory (to capture a broad range of participants’ perspectives without any preconceived notions or theories about these perspectives) [36]. The constant comparative method involves using inductive methodology to systematically generate theory from data. A combination of emerging codes from the data and pre-set codes generated from the FGD guide formed the root of the coding tree. The root pre-set code words for the coding tree were “roles of VHWs,” “VHW visits,” “VHW messages understood,” “VHW as community members,” “Questions asked VHWs,” “likes about VHWs,” “admire VHWs,” “VHWs working hard,” “encourage ANC and facility delivery,” and “dislike about VHWs.” All transcripts were analyzed through inductive theme analysis until all emerging themes are exhausted. Thereafter, the second author: SO analysed about 30% of the transcripts. There was an 85% inter-ratter agreement between the two coders (MAM and SO). Subsequently, the coding outcomes were shared through email on a power point presentation with co-authors: OS, BCO, BOO, IO and JA to consolidate codes into categories, and to identify overarching themes and sub-themes. A detailed report was provided alongside a presentation to Society for Family Health in April 2019.

## Results

### Socio-demographic, obstetric history, duration of contact with VHWs information

Fifty-eight (58) women beneficiaries of the VHW program participated in the study. Mean age was 25.1 (± 5.3) years old. Baganje North participants were older than those from Akko and Zange Wards. Over half (59%) of the participants have been exposed to secular education. Approximately half of the participants (51%), had no occupation. There was a higher representation of Muslims (69%) and the Fulani (36.2%) ethic group. All participants (100%) were married, 76% had between 1–4 living children. Within the last 12 months, 64% delivered at a health facility. Most of the participants (67%), have been in contact with a VHW for a minimum of 10 months (Table 3).

### Focus group discussion findings

**Fig 3:**
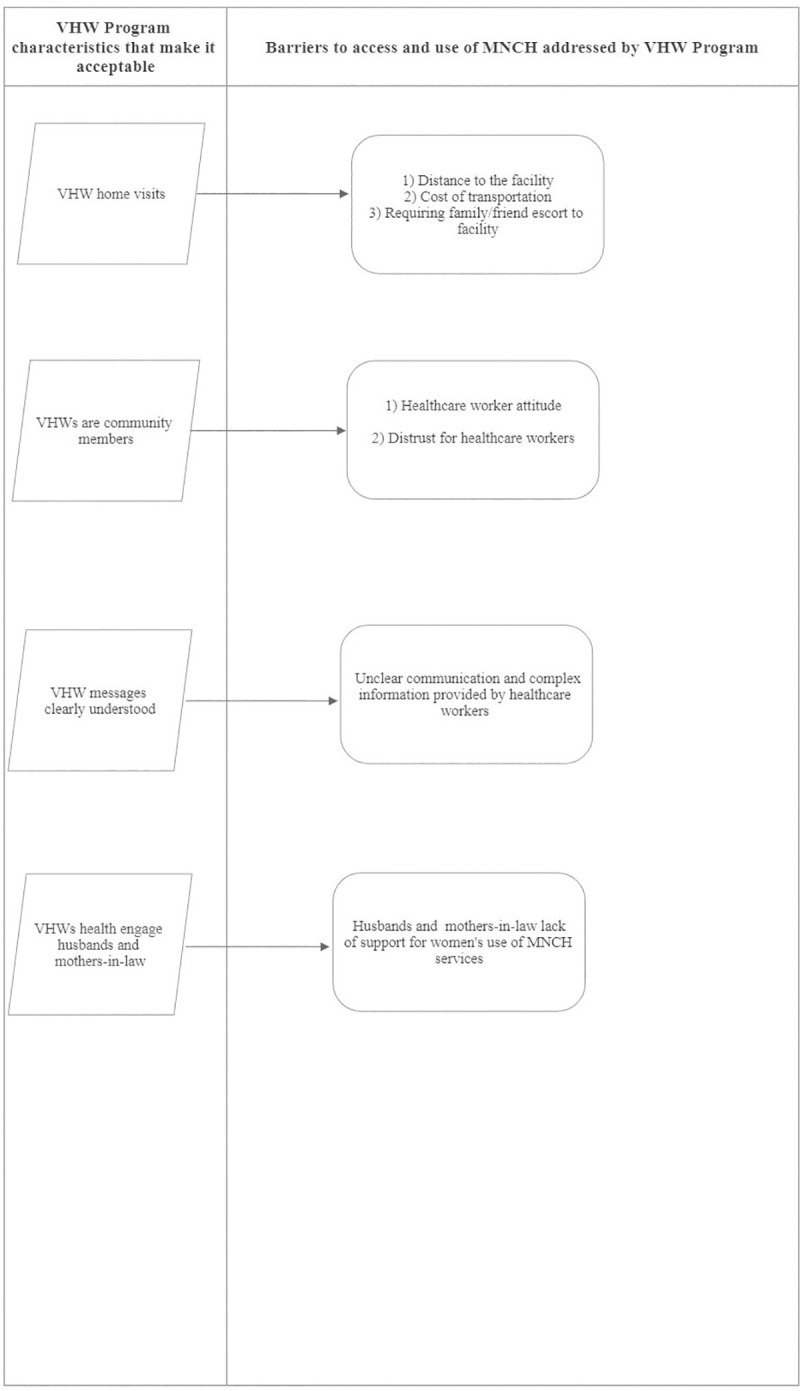
Acceptability of the Village Health Worker Program.

**Fig 4:**
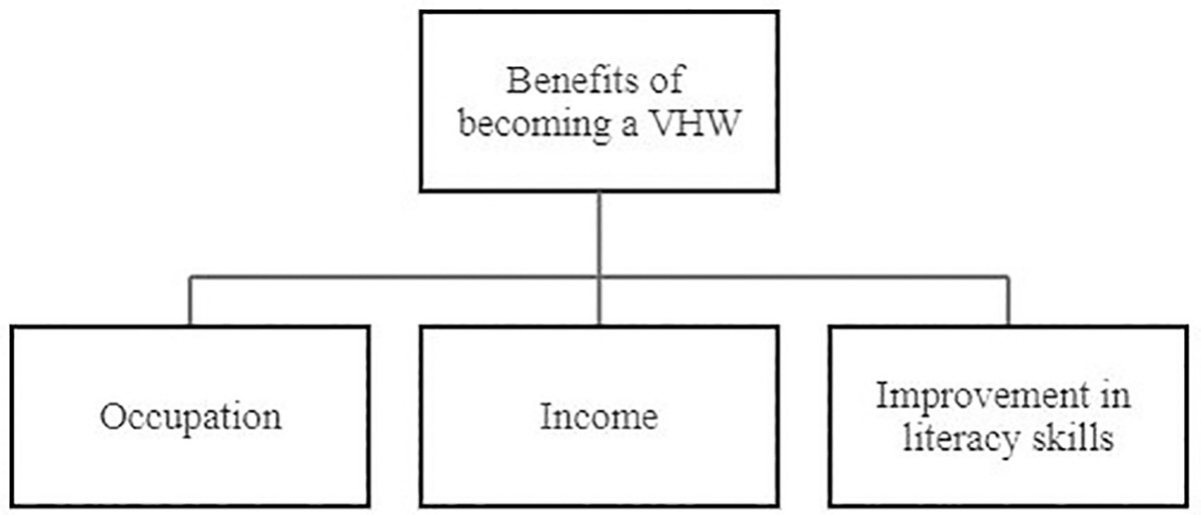
Social value of the Village Health Worker Program.

Eight (8) overarching themes emerged from the six focus group discussions: the first four themes centered around experience with, facilitators and barriers to facility delivery services, and recommendations on how increase the uptake of facility delivery services. While the last four themes centered on assessing the role, acceptability, social value, and recommendations for the VHW program. This paper will only be reporting the last four themes related to the roles and acceptability of the VHW program. Fig 3 displays the acceptability sub-themes and demonstrates how those sub-themes bridged barriers to the access and use of MNCH services. Fig 4 displays the sub-themes that emerged under VHW social value main theme.

### Roles of VHWs

VHWs roles included home visits to pregnant women to register them for antenatal care, to provide them with antenatal drugs, and educate them on healthy life practices during pregnancy and postpartum.

*VHW home visits improve access to maternal care*. Most participants appreciated the fact that VHWs visited them in their homes to register them for antenatal appointments and followed-up on them to ensure that they attended their clinic appointments. They also appreciated the fact that the VHWs distributed drugs (hematinic) to them in their homes, implying that their health was already being protected even before they visited the facility. Participants also stated that VHW MNCH promotional messages had changed their perception and attitude towards the use of facility services for antenatal, delivery and postnatal care.

*“I’m enjoying their* [VHW] *services*. *Sometimes even if you are reluctant in access facility services but after their visit*, *I just change my mind to avoid problem* [obstetric complications] *and visit the facility…”–* 23-year-old, Akko Home Group

Participants also mentioned that VHWs escorted them to the facility for antenatal appointments when they were in labor or when they fell sick. As some participants stated:

“*They* [VHWs] *escort one to facility during antenatal appointment and delivery*” 23-year-old, Akko Home Group

“*When they* [VHWs] *heard we are sick*, *they will quickly come and escort us to the facility*.” 35-year-old, Baganje North Group 1

Participants were immensely grateful to VHWs for the services they offered them.

*“They* [VHWs] *are doing their best*. *God should bless them”—*All Participants, Akko Home Group

Prior to the advent of VHW intervention within communities, many women go through pregnancy and childbirth without ever using facility services. However, the health education and mentorship of VHWs led many women to become enlightened and aware of the health benefits of facility MNCH services for the mother-infant pair, which participants believed has resulted in a substantial increase in the uptake of facility MNCH services.

*“Some women don’t even have the intention of visiting the facility for ANC*, *but because of their* [VHW’s] *persistent visits*, *they now go and when we go*, *we enjoy the facility visits because those drugs that we are given assists us a lot and protects us from diseases and make us healthy*.*”* 20-year-old, Akko Facility Group

*Health education by VHWs enhanced MNCH knowledge of women*. Participants in all the six groups clearly stated that they were happy with the health education they received from VHWs. Apart from educating them on the benefits of utilizing facility based MNCH health services, participants stated that VHWs educated them on the danger signs of pregnancy, personal and environmental hygiene, good nutrition, including disease prevention and health promotion living habits.

“We feel happy about them and they do tell us the importance of ANC and what will happen if you don’t go, they also show us some danger signs that we need to take note of like swelling of the body, bleeding, lack of blood, abdominal pain, severe back pain and many more, any time we notice this signs we should rush to the hospital.”

– 20-year-old, Zange Facility Group

*“…they* [VHWs] *give advice … the food you are expect to eat*, *the way to hold your child and the way you breast feed your child until he/she grow s up*.*”*

– 18-year-old, Akko Home Group

Some participants went on to describe how VHW messages had positively influenced the manner they fed their infants. They iterated that their infants with whom they practiced exclusive breastfeeding as recommended by VHWs, were healthier than infants with whom they practiced mixed feeding.

*“When I gave some of my older children traditional medicine* [when they were infants] *they got sick a lot but this girl I am only breastfeeding her*, *I don’t give her water and she don’t fall sick a lot*. *They* [VHWs] *have enlightened me and I am now aware of the difference between what I practiced before and now this girl*, *I didn’t even encounter any problem*. *Even when she is teething but with the previous kids*…*”*

– 35-year-old, Baganje North Group 1

#### Assessing the acceptability of the VHW program

VHW services and messages were clearly understood and accepted by the women because the VHWs were familiar community members and were therefore easy to relate and interact with. In addition, the VHW role created a platform for the development of personal relationship(s) between women and their respective VHWs. Most participants expressed immense satisfaction with VHW services.

*Familiarity with VHWs*. Most participants in all the six groups indicated that they were satisfied with the fact that VHWs were indigenous members of their communities. Many participants expressed that VHWs were either their friends, family members, housemates, or neighbors. Thus, they could freely associate with, and trust to confide in them. As some respondents stated:

*“We grow up together*, *I have elder ones youngers one and my mates my friends as VHW so I’m free with them*.”- 31-year-old,—Akko Home Group

*“We feel free with them* [VHWs] *because while we live in the same community with some*, *we live in the same house with others*.*–* 25-year-old, Akko Facility Group

Furthermore, though participants will not refuse the services of out-of-community VHWs, they preferred VHWs that were community residents. This is because community VHWs’ services were easily accessible on demand especially in case of an emergency. As some participants stated:

*“Honestly*, *we feel our community members are better* [to serve as our VHWs]. *We will not refuse if they* [VHWs] *are from other communities but those from our communities are better*.*–* 25-year-old, 3 FDs and 1 HD (AFG)

*Its better because if anything happen*, *we can call her* [the VHW] *at any time since we are in the same community*, *I don’t think it’s a problem because she’s from our community*.*”* - 20-year-old, Zange Facility Group

Furthermore, participants also expressed that they interacted freely with VHWs. Participants did not hesitate to ask VHWs questions related to the informational messages they delivered to them, and on issues concerning their own personal health.

*“Yes*, *we feel free to ask them* [VHWs]*questions about information they deliver to us*.*”*

- All Participants—Baganje North Group 2

“[I] *Ask her about the solution to my bleeding during pregnancy*, *she told me to go to the facility to get medication*.”

- 27-year-old, Baganje North Group 2

*Information conveyed by VHWs clearly understood*. Participants in the six focus groups strongly expressed that the information conveyed to them by VHWs was clear and comprehensible. When asked ‘if they understood the message of the VHWs’ all participants from two of the groups responded in the affirmative. Furthermore, to demonstrate their level of understanding of the health education they received from VHWs, participants iterated what they had learnt from those educative interactive sessions:

*“I understand their* [VHW’s] *message very well*. *They talk about hand washing and keeping our surrounding clean*, *avoiding stagnant waters around our homes then going to facility and types of foods to be eaten*.*”–* 34-year-old, Akko Home Group

“*They* [VHWs] *show us pictures of various classes of food and how these foods can be eaten*. *You don’t need to disturb your husband to buy you meat*, *fish but you can make use of beans which can serve the purpose*.”– 18-year-old, Akko Home Group

In addition, participants in all six groups expressed that they clearly understood the information and picture illustrations on the flip charts used by VHWs as teaching aid during health talks.

*“They* [VHWs] *use to show women during ANC*, *pictures of women with various complications*.*–* 23-year-old, Akko Home Group

“*Yes*, *we understand the pictures very well*.”– 20-year-old, Akko Facility Group

Participants also indicated that the information they received from VHWs was similar in content to the information they received from healthcare workers in the health facilities.

*“We understand every information the VHW conveys to us and there is no difference between the information we get from the provider in the health facility*.*”–*All Participants, Zange Home Group

However, some participants implied through their statements that they were more satisfied with VHWs’ strategy of delivering health educational sessions in comparison to what was accessible to them from facility healthcare workers. This is because while healthcare workers were permanently stationed in the facility and do not pay them home visits, VHWs enlightened them and explained pregnancy and other health issues to them within the comfort of their homes. Furthermore, VHWs were able to re-enforce their earlier message if they assessed in their subsequent follow-up visits that the educational advice they provided was not being practiced.

*“The difference between the information the VHW conveys and that of health providers is that*, *the VHW advices me and always visits me at home to ensure I practice it while the health providers only advise us in the facility*.”

- 30-year-old, Baganje North Group 2

Participants also valued and anticipated VHW visits because they had developed sociable relationships with their respective VHWs which has turned into a valuable companionship.

*“What I like about them*, *when they entered my house*, *I use to be very happy because of the health education they gave me*, *they use to encourage me and they use to explain a lot of things*, *those that we don’t understand*, *they make me more enlighten that is why I am interested in seeing them all the time because if they entered my house*, *my face is lid up*, *and I am happy because they came*, *we use to gist a lot and they calm me down even when I am over thinking*, *you will see that my anger will disappear and we will gist*. *That is why I am happy about them*.*”-* 30-year-old, Baganje North Group 2

*Satisfaction with VHWs*. Most participants expressed overwhelming satisfaction with the function of the VHWs and did not express any aspect of the VHW program in terms of MNCH services that they felt should be improved upon.

*“They are doing their best*. *I don’t think there is any area* [in maternal and infant health] *that we need any more help*.*”* - 23-year-old, Akko Home Group

Participants in all groups admired VHWs work ethics and expressed that VHWs were enthusiastic and hardworking considering they transverse remote areas to be able to reach women living there.

*“They* [VHWs] *visit ‘rugan Fulani’* [remote Fulani communities], *from one village to the other 3 to 4 times a week*. *Some places can only be access using motorcycles*. *They are suffering*.*”* - 31-year-old, Akko Home Group

#### Social value of the VHW program

This theme refers to the roles VHWs played within women’s social circle to enhance the use of MNCH facility services. VHWs intervened with husbands and mothers-in-law to allow pregnant women to access facility delivery services. Furthermore, women who qualify as VHWs seemed to improve their literacy skills.

*Awareness among mothers in-law*. Some participants mentioned that in the past, mothers-in-law did not support their daughters-in law to use facility MNCH services, but because mothers-in-law are now enlightened on the health benefits of MNCH facility services through VHW health education sessions, they now support their daughters-in-law to use MNCH services.

*“They* [mothers-in-law] *agree because they know the importance of facility delivery*. *When the VHW come for sensitization*, *they engage all of us like 10 women at the sometime*, *including the mother in-laws so; they don’t have any problem as regards to that*…*”* - 25-year-old, Akko Facility Group

*Awareness among Husbands*. VHWs also counselled husbands together with their wives during VHW home visits. They advised husbands to allow their wives attend antenatal appointments and to convey their wives to the facility for delivery at the on-set of labour. This is supported by participants quotes below:

*“Yes*, *she* [VHW] *advises me together with my husband*.*”* 29- year-old, Baganje North Group 1

*“He knows because the* VHW *use to visit house to house*, *they use to talk us about the importance of going to the facility*, *she advise us; she told him that when I am in labour*, *he should take me to the facility*, *hence he use her advice and allow me to come for delivery in the facility*.*”–* 29- year-old, Baganje North Group 1

Apart from advising husbands to support their wives to deliver in the facility, the VHWs were also able to convince husbands to allow their wives to attend their antenatal appointments.

*“*…*Sometimes even if the husband has issue with you attending facility the VHW have a way to convincing him into allowing you attend facility*.*”–* 23-year-old, Akko Home Group

*VHW literacy levels seemingly improve on the job*. Some participants admired the fact that when selected community members qualify as VHWs, their literacy level improved. Though basic reading and writing skills were among the requisite criteria to be selected and trained as a VHW, some VHWs made extra effort to improve their literacy levels on the job.

*“What I like is some of them* [VHWs] *don’t know how to write and read before*, *but now when they started the VHW they are able to read and write*. *Because when you don’t know how to read there is no way you can (recording inaudible)*.*”* - 30-year-old, Baganje North Group 1

Therefore, the VHW occupation was cherished and admired by participants and some of the participants aspired to be VHWs:

*“I love their [VHW’s] bags and I feel like becoming a VHW too*.*” -* 33-year-old, Baganje North Group 2

#### Recommendations for the VHW program

Participants recommended that the VHW program should be scaled up to other parts of the state that the program is yet to be implemented. They also recommended that VHWs should be offered capacity building trainings, and better terms of employment.

*Up-scaling the program*. Most participants when asked if they had more comments after the FGD discussions, they stated that they would like the VHW program to be scaled-up to other communities where the program is yet to be activated. This so that other women would also benefit from the services of VHWs with the intended consequence of reducing the prevalence of home deliveries and pre-term births in those communities.

*“There are areas that are lacking VHWs*, *so if it's possible they should also be given VHWs so that they too can benefit the way we are benefiting*. *It will also reduce the rate of home deliveries in such communities and also cases of woman with premature babies will be reduced*.*”* - 30-year-old, Baganje North Group 2.

*“I want their work to reach others*. *To expand their scope to reach others*.*”* - 23-year-old, Akko Home Group.

*Capacity building and financial support for VHWs*. Some participants recommended that VHWs should be given basic obstetric training that will enable them to assess the stage and progress of labor. This is so that women’s arrival at the facility for delivery will be targeted and timely.

*“…please help them* [VHWs] *with training on childbirth*, *because if we came to the hospital and if our delivery will be in 6 hours*, *we use to wait for a long period of time*, *with the health personnel*, *so if the VHW check she will be able to tell us when to go to facility*. *I want them to be trained so that they can help us in the community*.*”* - 35-year-old, Baganje North Group 1.

Some participants appreciated the services of VHWs to the extent that wanted VHWs to be employed either by healthcare facilities or the government, and be supported with transportation fee.

*“…I wish the government will employ her* [the VHW] *and I will also benefit from her*.*”-* 29-year-old, Baganje North Group 2.

*“if there is money their transportation should be paid*.*”* - 29-year-old, Baganje North Group 2.

## Discussion

VHWs roles in Gombe State included home visits to pregnant women to provide basic maternal care, health education and facility linkage. These roles were acceptable to women beneficiaries of the VHW program. The women believed that the VHW program had improved their health literacy and uptake of MNCH services. They also appreciated that the program provided VHWs an occupation, source of income, and an opportunity to improve their literacy skills. Participants recommended VHW program scale-up, VHW capacity building and better terms of engagement.

Participants appreciated the roles VHWs play in their lives as teachers, mentors, companions, and educators, and similar to findings from Nigeria, Burkina Faso and Uganda [37], they revealed no negative aspect of the VHW program and believed VHW interventions improved their uptake of MNCH services. The improvement in the uptake of MNCH services can be supported by the success of the VHW program: facility delivery uptake among VHW beneficiaries was 65%—over 2-fold higher than the state’s baseline facility delivery uptake of 27.6% in 2013 [35]. Facility delivery is usually higher (73%) in women with prior exposure to VHW messages than in women (56.6%) who did not receive VHW messages [38].

It can be deduced from the FGDs that there were four characteristics of the VHW program that made it acceptable: First, VHW home-visits, and their role in escorting women to the facility bridged three barriers to accessing MNCH services: distance to the facility [27, 29], transportation and facility service cost [27], and requiring family/friend escort to the facility [35]. Furthermore, considering over half (51%) of our study participants were unemployed and probably stayed at home as care providers, VHW home visits as reported from South Africa [28], provided women a service and a personal relationship with corresponding mental and psychological support. Evidence has shown that this type of VHW-woman personal relationship improved the uptake of VHW services [27]. In line with Jegede et al (2016) findings in Nigeria [37], our participants also appreciated the immediate physical access to VHW services.

Second, similar to findings in Ethiopia [9] and South Africa [28], VHWs were indigenous community members in which they served. This addressed the problem of healthcare provider disrespectful attitude and obliviousness to community norms and values that limit use of MNCH service in Gombe and other parts of Nigeria [28, 35]. Utilization of VHW services was sometimes reported as poor when VHWs had different cultures or languages than their recipients [27]. Furthermore, receiving VHW services from familiar community members gave women the confidence to trust VHWs and their informational messages [28], and the ease to ask them questions.

Third, the educational component of the VHW program was designed to meet the needs of the program beneficiaries because participants clearly understood VHW messages and teaching tools (flip charts). It is vital that health information is simplified to the intellectual level of the women so they are not confused [39]. Furthermore the clarity of VHW information was augmented with the delivery of culturally appropriate VHW services [10–12]. Fourth, the VHWs’ intervention has been successful in getting the buy-in of mothers-in-law and husbands to support women’s MNCH visits [40, 41], who happen to be major stakeholders in women’s access and use of MNCH services [39, 42].

The acceptability of VHW is further illustrated by the fact that participants advocated for VHWs to be trained in basic obstetric care so that the VHW will be able to assess their stage of labour and enhance their timely arrival at the facility. This could imply that women preferred to be cared for by VHWs in their homes rather than being in the facility with healthcare workers even during labour. This could mean that women were either not satisfied with the quality of care in the facility [43], and/or they experienced mistreatment from the healthcare workers [44]. Dissatisfaction with facility services could also be the reason some women delivered at home despite their overwhelming acceptance of VHW’s advice on the importance of facility delivery.

Furthermore, participants suggested that VHWs should be employed either by the health facilities or by the government and offered transportation fee. Their suggestion aligns with fact that VHWs’ job dissatisfaction were usually related to none or inadequate financial compensation and/or career path [27, 37, 45, 46]. Countries with successful VHW programs (Ethiopia and Bangladesh) have been supported and sustained by their respective governments [47]. However, the three main characteristics of the Gombe VHW program that can enable its up-scale and sustainability, included: 1) VHW ongoing management and supervision. 2) the program’s compatibility with community norms and support by community members. 3) Government support [48].

Lastly, the success and commendations of the VHW program transcended its primary objective of increasing the uptake of MNCH services and alleviating the healthcare provider insufficiency in Gombe State [31]. The VHW program engaged unemployed women as VHWs thereby providing them with an occupation and income and subsequently contributing to broader developmental outcomes [49]. The fact that the VHW program was acceptable in Gombe State, one of the states with the highest maternal and under-five mortality rates and very low uptake of MNCH facility services in Nigeria [31–33, 35], demonstrates the feasibility of scaling the VHW program to other parts of the country. Just like the Gombe State VHW program, VHW programs for low resource settings should be tailored to accommodate the socio-economic, cultural, and intellectual context of the communities it is meant to serve and be implemented with a financially viable sustainability strategy.

### Study limitations and strengths

The fact that all participants regardless of their wards of residence (with either maximum, average, and minimum uptake of facility delivery among the 57 VHW wards) found VHWs acceptable, gives our data some level of validity and indicates that the uptake of facility delivery services was probably unrelated to the acceptability of VHWs. Typical of qualitative studies, our findings are not generalizable to the whole of Gombe State. However, many Gombe residents live in the same rural areas as our participants and face the same challenges with access and use of maternal health services.

Our study is not without limitations. First, the fact that participants were invited to attend the FGDs by VHWs and had inter-personal relationships with them could have biased their responses. However, collecting information directly from beneficiaries of the VHW program gave us invaluable benchmark of assessing the value of the VHW program to those it was targeted to serve. Second, our findings were limited to the perspectives of the women beneficiaries of the program. FGDs, key informant or in-depth interviews with VHWs and other community members may have provided the opportunity to triangulate data for further validation. Third, translating and transcribing FGDs conducted in Hausa may have led to some loss of information.

## Conclusion

The Gombe State VHW Program was the first government led VHW Program in Nigeria, and women beneficiaries of the program found it acceptable and believed the program improved their health literacy and uptake of MNCH services. Our findings further contribute to body of evidence that support the important role VHWs services play in improving MNCH indicators and alleviating healthcare worker insufficiency in resource poor settings.

## Supporting information

S1 File Focus group guide (Hausa version). (PDF)

S2 File Focus group guide (English version). (PDF)

S3 File Sociodemographic information of participants. (XLSX)

S4 File Transcripts of focus group discussions. (PDF)

S5 File COREQ guidelines. (PDF)
