# Supplementary material for: Assessing the acceptability of village health workers’ roles in improving maternal health care in Gombe State, Nigeria a qualitative exploration from women beneficiaries
Source: PLoS One. 2020 Oct 22;15(10):e0240798. doi: 10.1371/journal.pone.0240798 (PMC7580965; doi:10.1371/journal.pone.0240798)
Supplement: S1 File — (PDF) [file pone.0240798.s001.pdf]

Appendix IV B: Focus Group Moderator Guide (Translated in Hausa)

Tattaunawa Akan Abubuwan Dake Taimakawa, Da Abubuwan Dake Hana Ma Mata Masu Juna Biyu Waddanda Suka Ji Fadakarwan 'Village Health Workers', Zuwa Asibiti Su Haihu A Jahar Gombe

(Tattaunawa – da mata da suka samu fadakarwa daga 'Village Health Workers' kuma sun haihu a cikin shekara daya da wuce November 2017 – October 2018)

Before the group begins, conduct the informed consent process, including compensation discussion.

I. Gabatarwa (Minti 5)

Inna kwanan ku? Sunnan na __________. Ni zan jagoranci wannan tattaunawa da zamuyi yau. Za muyi kamar awa daya da rabi da ku. Zamu tauki wannan tattaunawa da zamuyi a casstte, kuma zakuga ____________ tana zauna tana rubutu a kan abubuwan da muke fada. To wannan domin mu tabbabtar cewa duk abuda muka tattauna a nan an rubuta, amma ba za a rubut sunnayen ku ba.

Zaki iya ki fadi sunnan da kike so a kira ki dashi a wannan tattaunawan da zamu yi (sunnan karya, ko wani suna daban)
To yansu sai kowace ta fadi sunnan da take so a kira ta dashi a wannan tattaunawa da zamuyi. Daya bayan daya Sunnan da kika zabama kanki, Wane irin abinci yafi miki dadi?

Tabbayoyi: in bam ai tabbaya, sai mu fara tattaunawa. (Begin recording)

Facilitator to speak into the tape recorder: and mention the following: **<u>Date, venue, time of FGD i.e ward and local government area and the participants (home or facility delivery group)</u>**

II. Ainihin Tattaunawa

Zuwa asibiti domin haihuwa (Minti 10)
Za mu fara da yanda kuke zuwa asibiti

1. Na farko, ku bamu labarin yanda kuke tasowa daga gidagen ku ku je asibiti. <u>Karin Bayani</u>: Kuna hawa moton haya, ko machine, ko da kafa? Asibitin nada nisa daga gidan ko? Kuna biyan kudin mota ko kudin machine?

2. Maigidan ki meye Ra'ayin shi game da Kiji asibiti ki haihu? <u>Karin Bayani:</u> Me kike ganin yake za maigidan ki ke so ki ji asibiti? Me kike ganin yake sa maigidanki baya zon ki haihu a asibiti?

3. Uwar mijinki meye ra'ayinta game da kiji asibiti ki haihu? <u>Karin bayani</u>: Me kike ganin yake sa uwar mijinki ta ke son ki haihu a asibiti? Me kike ganin yake da uwar mijinki bata zon ki haihu a asibiti?

Ra'ayin ku akan haihuwan asibiti (Minti 25):
Yanzu za mu tattauna akan kullawan da kuke samu a asibiti in kun zo haihuwa

4. Da kuka zo asibiti ki haihu, wane irin kullawa kuka samu? In baku taba haihuwa a asibiti ba, ki bamu labarin wasu da suka baku game da irin kulla da suka samu da suka je haihuwa a asibiti.
   <u>Karin bayani:</u> kukan samu kulla a nan take, ko sai kinta jira? Me kuke ganin dalillin dogon jira a asibiti? Me kuke ganin za'a iyya yi a gyara wannan matsala? Mallaman asibiti sun karrama ki?

5. Meye bambanchin haihuwa a gida da haihuwa a asibiti? <u>Karin bayani:</u> meye babbancin kullawan mallaman asibiti da na anguwan zoma?

6. Wanne mallaman asibiti kuka fi so ta karbi haihuwan ku? Mace ko namiji? Karin bayani: Meye dalillin zabin ki? In kin fada wanda kike zo ya karbi haihuwan ki a asibiti, mallaman asibiti suna biya miki bukarki?

7. Me kuke gannin sai taimaka muku zuwa asibiti ku haihu? Karin bayani: asibiti ya dawo kusa da gida ki, ko a gyara asibitin, ko haihuwa ya zama kwauta

8. Me kuke ganin sai taimaka ma sauran mata su zo asibiti su haihu?

Ra'ayinku akan VHWs (Minti 40)
Yanzu zamu tattauna akan ra'ayin ko akan VHWs

9. Meye ra'ayin ku akan fadakarwan da VHW suke yi muku? Karin bayani: kuna jin dadin fadakawars da suke muku?

10. Wane bangarorin ciki da haihuwa VHWs basa taimakon ku da su kuke so su su dinga taimaka muku da su?

11. Ya kuke ganin VHWs za su iyya haduwa da wasu mata kamarku wadanda kuke ganin suna bukatar fadakarwan da VHWs suke muku?

12. Kuna ganin bayanen da VHWs suke muku in zu ziyarce ku a gidagenku? Karin bayani: meye bambancin fadakarwa da VHWs suke muku da irin fadakarwa da mallaman asibiti suke muku?

13. Kuna gane hotunan da VHWs suke nuna muku lokacin da suke fadakar daku?
Karin bayani: kuna ganin irin waddanan hotunan a asibiti? Meye babbancin the VHW da na asibiti?

14. Kuna sake jiki ku tambaye VHWs abubuwan da baku gane ba a kan fadakarwan da suke muku?
Karin bayani: Wane irin tambayoyi kuke tambayansu? Wane irin tambayoyi ke muku wahalan tambayan su?

15. Ziyarar da VHWs suke muku ya canza ra'ayin ku akan zuwa asibiti ku haihu? Karin bayani: ta ya VHWs suka canza ra'ayin ku?

16. Meye ra'ayin ku da cewa VHWs mutanen anguwanku ne? Karin bayani: wane matsaloli kuke ganin wannan ke haifarwa? Karin bayani: kuna jin dadin cewa VHWs yan anguanku ne?

17. Me yake baku sha'awa akan VHWs?

18. Me baya baku sha'awa akan VHWs?

III. **Rufe Taro** (10 min)

Za mu rufe wannan taro da tabbaya in akwai wani bayyani da kuke so ku kara akan wanda muka yi. (comment: allow time for general discussion). In akwai masu tambaya sai suyi yanzu (allow for questions, and give answers if necessary)

Muna matukar godiya da kuka zo daga gidagen ku kuka tattauna damu; Allah ya saka muku da alheri ya kuma maida ku gidagenku lafiya. (Comment: Issue their refreshment immediately)
