# Supplementary material for: Assessing the acceptability of village health workers’ roles in improving maternal health care in Gombe State, Nigeria a qualitative exploration from women beneficiaries
Source: PLoS One. 2020 Oct 22;15(10):e0240798. doi: 10.1371/journal.pone.0240798 (PMC7580965; doi:10.1371/journal.pone.0240798)
Supplement: S2 File — (PDF) [file pone.0240798.s002.pdf]

Appendix IV A: Focus Group Moderator Guide

Study Title: Barriers and Facilitators to the Utilization of Facility Delivery Services for Beneficiaries of the Village Health Worker Program in Gombe State

(Focus Groups- for Village Health Worker Program Beneficiaries Who Delivered Babies in the last 12 months November 2017 – October 2018)

Before the group begins, conduct the informed consent process, including compensation discussion.

I. Introduction (5 minutes)

Good Morning/Afternoon/Evening: My name is_________________. I will be your discussion leader for today. We are going to spend 1 hour and thirty minutes talking about issues that will help us better understand the why some women use facility delivery services and why other women do not use those services. We also want to get your views on the VHW program. You will also notice that there is an observer who will be taking notes of the conversation. This is being done to help us accurately track the conversations.

You can indicate what name you want to be addressed with during the discussion (you can use a nickname or an alternate name).

Let's introduce ourselves with names/nicknames we want to be addressed with. Please share with the group: Fake name, Your favorite food;

Questions: if there are no further questions, we would like to begin the discussion. (Begin recording)

Facilitator to speak into the tape recorder: and mention the following: **<u>Date, venue, time of FGD i.e ward and local government area and the participants (home or facility delivery group)</u>**

II. Main Discussion

Access to Facility Delivery (10 minutes)

Let's talk about getting to the health facility

1. First, tell us a bit about how you travel to the health facilities. <u>Probes:</u> Are the facilities too far from where you live or hard to get to? Do you have to pay for transportation?

2. How does your husband feel about you delivering your baby in the facility? <u>Probes:</u> why do you think he supports you to deliver in the facility? why do you think he doesn't support you to deliver in the facility?

3. How does your mother-in law feel about you delivering your baby in the facility? <u>Probes</u>: why do you think he supports you to deliver in the facility? why do you think he doesn't support you to deliver in the facility?

Views and Experiences about Facility Deliveries (25 minutes):

Let's talk about the care you get when you come to deliver in the health facility

4. How did you feel when you came to the facility to deliver your baby? If you have never delivered in the facility, tell us the experience of others. <u>Probes:</u> what usually happens- are you seen right away, or do you have to wait long? why is that the case? what do you think we should do to improve this?
   Do you feel that the staff treated you with respect and value your point of view?

5. How is delivering at the facility different from delivering at home? <u>Probe:</u> compare the services you get from the TBA to the services you received from a health care provider at the facility.

6. Do you have any special religious or cultural requirements- such as needing to be seen only by a female healthcare provider? How has the staff responded to these needs? <u>Probe</u>: Overall have you found the staff to be welcoming?

7. What do you think can be done to facilitate you to deliver in the health facility? Probes: ease access, improve quality of services, user fees

8. What do you think can be done to facilitate other women to deliver in the health facility?

Acceptability of the VHW Program (40 minutes)
The next part of the focus group looks the views and experiences of participants with the VHW Program. Let's talk about your experiences with VHWs in your area.

9. How do you feel about the VHWs? <u>Probe:</u> How do you feel about the services they provide to you and other women?

10. What aspect of pregnancy and childbirth are the VHW not helping you with, that you would you like them to help you with?

11. How can VHW reach more women like you?

12. Do you feel you understand the information the VHW conveys to you when she visits you? <u>Probes:</u> How is the information she coveys to you different from the information you get from the providers in the health facility?

13. Do you feel you understand the flip chart the VHW uses for health education?
<u>Probes:</u> do you see these kinds of charts in the health facility?
How are the charts in the health facility different from the ones you see with VHWs?

14. Do you feel free to ask VHW questions about the information she delivers to you? <u>Probes:</u> what kind of questions can you ask her? What kind of questions are you not able to ask her?

15. Has VHW visits to the community changed your perception of facility deliveries?

16. How do you feel about the fact that the VHWs are members of your community? <u>Probes:</u> what problems do you think is associated with this? Why is that a good thing?

17. What do you like about the VHW program? 18. What do you not like about the VHW program?

III. Closing (10 min)

We would like to finish our discussion by asking if there are any suggestions you have for us or anything you would like to say in closing. (comment: allow time for general discussion).
We would like to thank you for spending time with us, we appreciate all that you told us.
(Comment: Issue their refreshment immediately)
