# Supplementary material for: Assessing the acceptability of village health workers’ roles in improving maternal health care in Gombe State, Nigeria a qualitative exploration from women beneficiaries
Source: PLoS One. 2020 Oct 22;15(10):e0240798. doi: 10.1371/journal.pone.0240798 (PMC7580965; doi:10.1371/journal.pone.0240798)
Supplement: S4 File — (PDF) [file pone.0240798.s004.pdf]

Focus Group 1

TITLE: Focus Group Discussion with mothers who delivered their babies in the facility

DURATION OF RECORDING: 1Hour 33 minutes 04 seconds
NUMBER OF PARTICIPANTS: 7

Acronyms
ETS- Emergency Transport Services
VHW- Village Health Worker
ANC- Ante Natal Care
TBA- Traditional Birth Attendant
FGD- Focus Group Discussion

[Beginning of recorded material]

Probe: Please speak loudly so as the voice will be recorded in the radio.
All participants' response: yes

Q1: Now we are going to talk about access to facility for delivery, how you travel to the facility to deliver your babies? First, tell me about how you travel for facility delivery, what means of transportation do you used in travelling to the facility?

Response: we use to go to the ETS; He will take us and bring us to the hospital. Then we came and delivered in the Hospital.

Probe**:** where is your community or your ward?
Response: I live in xxxx community; my house is far but not too far.

Probe: Far from this facility? Did you deliver in this facility (referring to the facility where the FGD is taking place)?
Response: Yes, I gave birth in the facility here at xxx (referring to the facility where the FGD is taking place)

Probe: then, who next? Do you pay for transport for coming to the facility?
Response: No is the ETS that use to bring us here.
Response: If I started feeling labour, we use to call the ETS because we have their numbers, he will bring us to the hospital for delivery.

Probe: In your last delivery, what do you use in transporting to the facility?
Response: The ETS conveyed me to the facility.

Probe: So, he is the one that brought you to the facility?
Response: Yes

Probe: Apart from the ETS, apart from his car, what other means do women in labour use for transport to the facility?

Response: No, he is the only car we use in coming to the facility.

Probe: What of you: what method of transportation did you use in conveying yourself for hospital delivery?

Response: Car

Probe: what do you mean; did the ETS bring you to the facility?

Response: Yes, the ETS conveyed me to the facility.

Probe: What of you xxx?

Response: when I started feeling the labour, we call the ETS and I was conveyed to the facility.

Probe: is your place of residence far from the facility?

Response: is not too far.

Probe: what of you xxx?

Response: it was the ETS that conveyed me when I started feeling pain; the ETS took me to the hospital.

Probe: is the facility far from your place of residence.

Response: no is not far.

Response: when I started feeling labour, I called the ETS and they came and conveyed me to the facility, and I delivered here.

Probe: is your house far from the hospital?

Response: Yes, is a bit far.

Probe: What of you the xxx?

Response: when I start feeling labour pain, I took the ETS number and they call him and they conveyed me here, xxx hospital.

Probe: Is your house far from the hospital?

Response: is not too far.

Q2: How does your husband feels about you delivering your baby in the facility? How does your husband feel about it?

Response: My husband supports me to deliver in the facility because there are diseases that people contracted after child delivery, that is why he wants me to deliver in the facility.

Probe: What of you xxx: What is your husband's opinion?

Response: My husband is in support because there are diseases, high blood pressure. Those that are far from the facility end up not delivering in the facility mostly result in complications e.g. if the blood pressure is high; there is no way of treating it at home, that is why he supported of facility delivery and he said is good for us to come and deliver our babies in the facility.

Probe: What of you xxx: how does your husband feels about delivering your baby in the facility?

Response: He do not want to stop me; he is in support.

Probe: Do you mean he supports you?

Response: Yes, he is in support.

Probe: xxx, how does your husband feel about coming to the facility for delivery?

Response: He is support because over bleeding occurs often when women delivered, and the facility readily administers injection…. (Murmured inaudible words)

Probe: What of you xxxx, do you have something to add?

Response: My husbands supported because there is immunisation administered in the facility for preventing us from getting sick.

Probe: continue… are you done?

Response: Yes

Probe: xxx do you want to add to what she said, about how your husband feels?

Response: My husband is in support of facility delivery because when I started experiencing labour, he got worried and quickly called the ETS and they conveyed me to the facility. That is the reason why he allows me, and it is important to give birth in the facility to avoid complications like over bleeding and other illness. We could get treated in the hospital.

Probe: What of you xxx?

Response: Bleeding can occur during childbirth and is normally stopped by administering an injection. If a woman gave birth, there could be high blood pressure, and the facility will treat such problem.

Probe: So, what do you think makes your husband to support you to go the facility? Is there anything, or any awareness that makes them comfortable with you going the facility?

Response: Because he is aware that the facility delivery is good and is important, that is why they are comfortable with delivering our babies in the health facility?

Probe: How did he become aware of the importance of facility delivery?

Response: He knows because the VHW use to visit house to house, they use to talk us about the importance of going to the facility, she advise us; she told him that when I am in labour, he should take me to the facility, hence he use her advice and allow me to come for delivery in the facility.

Probe: So, does she advise you together, with your husband?

Response: Yes, she advises me together with my husband.

Probe: is there any other person that wants to contribute?

Response: The reason why he allow me is because he is aware of it importance, if I gave birth at home, and complications occur, there is no way of treating it e.g. high blood pressure, he cannot administer the required injection to prevent it. If there is bleeding, he cannot treat it, that is why he believes that facility delivery is a most.

Probe: is there any other person that wants to add more reasons?

Response: Sometimes complications occur after delivery or during labour and are treated with injections in the facility. That is why he allow us

Probe: Is there any woman with more explanation?

Response: The reason why my husband is ok with it is, if child is not breathing immediately after home delivery, they are often buried, but in the facility, they are aware whether he is breathing or not. When we attend ANC, we were informed to avoid home delivery and always come to facility for our delivery.

Q2: **Well done, now we have heard about how your husband's feels about facility delivery. Ok, now I want you to tell me about how your mother in-laws feel about delivering your babies in the facility?**

Response 1: tell us more about your mother in laws opinion!

Response 2: She is in support; she is aware, and she is really happy.

Probe: what kind of help does she render?

Response…. (silent).

Probe: Before you remember, can you, tell us about what your mother in-law opinion is on delivering your baby at the facility?

Response: My mother in-law gets worried when I am late for my ANC, she use to insist that I go to the facility. Because if I am ill, or the foetus wasn't in the right position, they will be able to examine it, that is why she instead I go all the time.

Probe: what is the opinion of your mother in-law about your delivery at the facility?

Response: She is in support of me to deliver in the facility so as to check my wellbeing.

Probe: Is there anybody with more explanation on what mother in-laws feel about your facility delivery?

Response: Yes, my mother in law is in support of my facility delivery because if umbilical cord is cut there is a lotion that is being applied on the umbilical cord. Facility visit is quite important to her because she know that I will be examined thoroughly.

00.14.01 PROBE: We are progressing; we are going to the next session. Views and experiences about facility delivery, we have talked about how you access the facility. Now, let us talk about the care you get in the facility

PROBE: did you all gave birth in the facility

All participants' response: Yes

Q3: How do you feel when you came to the facility to deliver your baby? What was the type of service rendered to you? I am sure all of you have been attended to, so I want you to tell me the kind of service rendered to you? I am starting with you: tell me about what you experienced when you came to deliver your baby?

Response: First, when I came, she checked my blood pressure; so as when it is high, she will give me a medicine that will lower it, and she also advises me. When I was about to deliver she stayed by my side throughout, she examine my health, she check on how far due for delivery I was, sometimes it was an immediate delivery but other times I had to wait a little, she use to pray for me.

Probe: tell us about your own experience during childbirth in the hospital

Response: When we came to the hospital, I lay down and she examined me.

Probe: Tell me about what she was done to you when you came to deliver your baby or didn't, they assist you?

Response: Yes, they did.

Probe: Now tell me about the assistance giving to you; tell me about everything that they did for you?

Response: (No response)

Probe: Before she remembers, can xxx: tell us about the assistance rendered to her during childbirth?

Response: The truth is I was well taken care of, especially during this baby's birth (Pointing at her baby). We came to the facility at about 3.00 o. clocks, she examines my blood pressure, and she said I must wait for a bit, before I deliver. She checked on me from time to time, she also prayed for me, for safe delivery, she really assisted me, I even told her that she is lie a mother to me because of how she took good care of me.

Probe: Will someone else tell me about her experience when she came for facility delivery?

Response: when we came to the facility, I was reluctant to climb the bed, she insisted that I climb because If I gave birth on the floor, my child will contract many diseases, so I lay on the bed and deliver my baby. They prayed for me until I gave birth.

Probe: What of you xxx? Tell us about your own experience when you came for facility delivery.

Response: When I came, they took good care of me, they examine my blood pressure, we struggled together until I gave birth, and they really assisted me.

Probe: is there anyone with more information on her experience during childbirth?

Response: When I start experiencing labour, I was conveyed to this hospital at about 3:00 a.m. I met the nurse on night duty and she took care of me, she stayed close to me all night she didn't move away until I deliver my child, she cleaned the baby up and put him on my chest. She kept checking and examining me, and I told her that she is more than my mother, I really appreciate her, she assisted me as expected and I am grateful.

Probe: So now everybody has told me about their experience during facility delivery.

All participants' response: Yes.

Probe: I want to continue with my question, when you came for delivery in the facility, did you get immediate assistance, or did you have to wait or waste time?

Response: No there was no waste of time; I met the nurses in the facility.

Probe: Did you have to wait for them?

Response: No

Response: In my own case, it was on Saturday, the person on afternoon duty was not yet around. They called her, and she was arrived quickly before an hour.

Probe: So, when you came, she was absent, but she eventually came and attends to you?

Response: Yes

Probe: What of you xxx? did you wait before they attend to you or did, they gave you immediate assistance?

Response 1: I was attended to, immediately. I met them and they attend to me.

Response 2: I met them in the facility, and they attend to me without any waste of time. She showed me were to lie down.

Probe: is there anyone that wants to share more on their experience?

Response: I met them when I came, we sat and prayed.

Probe: Is there any other experience?

Response: I came around 3.00 am and I met the nurse, she immediately took me to the labour room, and laid me on the bed. She first checks my blood pressure and instructs me to lie on the bed.

Probe: So now none of you experience a waste of time?

Participants' response: Yes.

Probe: So now we are going for the next question, is there any one amongst you that delivered at home?

All participants' response: No.

Probe: So, all your babies are delivered in the facility?

All participants' response: Yes

Q4: **Tell me, how is delivery at the facility different from delivery at home? It doesn't have to be answered by the woman that gave birth at home only, I am sure we are all aware of the differences and I want you to tell me those differences? Who is starting?**

Response: The difference is, in the, sometimes the child delivered is covered with leather (amniotic sac) like a ball. If it is in the facility, the sac will be slit immediately, to enable the child to breathe but if it is at home it will be difficult for them to realise, and the infant may eventually die. The injection given at the hospital use to help increase women's wellbeing and the advice they use to give us.

Probe: Any other opinion?

Response: There is a difference, when I gave birth to my older son at home, I bleed a lot, I lost conscious, nobody understand, they just gave me some herbs to drink to stop the bleeding but when I came deliver in the facility; they gave me 3 tablets (misoprostol) and it stops the bleeding, that is the difference. They use to check on my blood pressure, check me and my baby's well-being and if my delivery time is near, which we can't know at home. That's why, the main difference, we use to get well in the facility.

Probe: I want to hear your view xxx?

Response: Well the difference between home and facility delivery is when I gave birth at home, then, we were not exposed, I bleed and lost consciousness, and I had to be cleaned unconsciously in the toilet. When I came for facility delivery, in the next pregnancy, I didn't encounter any problem. When I gave birth, they gave me the three tablets (Misoprostol) and I took it.

Response: The difference is when we gave birth at home, we will not get proper care while we get proper care in the facility, and we don't get medicine at home.

Response: The difference between facility and home delivery. Firstly, when we gave birth at home, I can encounter problem, and nobody is going to help me. If I over bleed

or get stomach-ache, nobody will be able to help me. But in the hospital, they will take care of me and give me 3 tablets (misoprostol) and they will tell me not to bath my baby until after 24 hours.

Probe: Is there any other views on the difference between home and facility delivery? Is there more information?

Participants' response: No.

Probe: So, what is the difference between the service of TBA and the facility service?

Response: The difference between TBA is always advising us on ANC and facility delivery, but the nurse conduct ANC, and examine us only in the facility, the TBA ensure that we go to the facility for ANC, that we are clean. ….. (Inaudible) and eat healthy food and so on. So that is their difference.

Probe: So, you mean the VHW are referred as the TBA?

Participants' response: Yes.

Probe: So, is there any difference the TBA (the VHW in their case) and the facility nurse?

Response: there is a difference. The TBA use to advise me to go to the facility so as the nurse will examine me and check my health. The TBA visits me always and that is her duty and she advise me.

Probe: is there is any opinion?

Response: TBA use to visit house to house and advise us to come to the facility, and when I am in labour the TBA will bring me to the facility and check that I am ok.

Response: more information on the difference between TBA and the nurses. The TBA use to visit us and teach us how to clean, while the nurse, when we met, they use to check us, and our blood pressure and they lower it. (Inaudible record because of children noise)

Probe: is there any different view?

All Participants: No

Q5: **What gender of nurse or facility personnel do you prefers, male or female? I know that we all have our preferences during delivery, and I would like to know more of your preferences?**

Response 1: I prefer a female because she is aware of the pain in childbirth, labour pain, I prefer female because she will take care of me.

Response 2: I am ok with whoever I met, because if I am to prefer a female and eventually met a man in the facility, I cannot stop him from attending to me. So I am ok with what I found in the facility.

Response 3: I like both male and female because they have attended to me during my delivery. When I gave birth to my first daughter in ____, it was a male that attended to me but now it is a female that attended to me.

Probe: But who do you prefer?

All participants' response: Both of them.

Probe: What of you xxx? Who do you prefer?

Response: I prefer a female because she knows the pain of childbirth and she knows how to take care of me. Then I know she is a woman; I am not going to hide anything from her will not be afraid.

Probe: Is there other reason apart from the one you mention?

Response: like the popular Hausa saying goes, I don't mind either of the sex I met, I am ok with whatever I met, I just want someone that will assist me, but men do try.

Probe: So, you don't really have a choice, but you said men are trying, so have any man attended to you before?

Response: No, a man has never attended to me, but I have seen the way they attend to others like my co-wives that is, my husband's brother's wife and friends. I used to see how they are trying.

Probe: So, who did not talk? Please add more information.

Response: When I came to the facility, I was taken care of. Then I am happy. I used to meet male personnel and they are helpful.

Probe: So, who do you prefer?

Response: Male.

Probe: Why?

Response: Because they are really taken care of me.

Probe: So, you think he took care of you more than the female?

Response: Yes.

Probe: So, all of us have spoken, right?

All participants' response: Yes.

Probe: So please tell me, does the health personnel met during childbirth, are they performing their duties, are you happy with their performance, and are your expectations met? Did they help you out?

Response: Yes, we are satisfied because if I came to the facility, and they check my health and my blood pressure, then I am satisfied. Because I came to the hospital because of her and she was really helpful and…

Probe: any other additional information?

Response: If I left my home and she visited me, and I seek for help then I am happy.

Probe: I meant the health worker?

Response: If she helps me with testing then I am satisfied.

Probe: I know but, when you came, did they render all the assistance you need?

Response: Yes, they help out.

Probe: Did they treat you well?

All participants' response: Yes

Probe: So, you didn't witness any form of maltreatment or bad comments?

Response: No form of bad treatment.

Probe: So, everybody has agreed that they have been treated well. Is there any contrary view? Please feel free to express yourself.

All participants' response: No bad treatment, they treat us well, and give us all assistance that we seek.

Q6: What do you think will support you and facilitate more women to use facility for delivery? Things that will make you interested in coming to the hospital more? What do you think will make you come more?

Response: What makes me like delivering in the hospital, first when I came she will assist me, she will inject me, after I left, they use to give me gift, they gave me bag, and soap, umbilical cord lotion, etc. and pants, that is why my interested is sustained in always coming to the facility for child delivery.

Probe: So, is because of her the gift?

Response: Yes, and the health check-up.

Probe: Is there any other view, things that will make you come more often and deliver in the hospital? Like what and what?

Probe: Like those things that will make those that don't come to the facility, what do you think can be done to make them come? Since all of you have been visited by the VHW, right?

All participants' response: Yes

Probe: So, what do you think will draw the attention of those that are not coming to facility to come to the facility?

Response: In my own opinion what will make those that are not coming to have interest. We use to come to the hospital, we use to tell them that we got essential medicine, if there are medicine with other gifts given to us, when they saw it, it will make them interested in coming to hospital, like when we told them we use to be healthy now, unlike before, when we deliver at home. Even ANC we don't use to attend before, we use to attend once only, but now we are interested in coming for regular ANC, we

want them to examine our health and blood pressure in other to be healthy. So, if others see what is happening, they use to get interested and say that a woman went to the facility and that is how to make them become interested.

Probe: Is the medicine given at free of charge?

Response: Yes, it is.

Probe: Is there any other person with more explanation?

Response: The reason why I use to come to the hospital and get interested, they use to give us mosquito net to protect our health and our baby's health. To prevent malaria fever, also we are injected at the health facility in other for us to be healthy

Probe: Now all of you are telling us about your own opinion, on why you go to the facility right? **All participants' response**: Yes

Probe: Then I want to know, your friends, or family or house members that don't come to the facility, what can we do to make them start coming to the facility?

Response: When I went to the facility, I came back then my friends ask me about what I was given that use to make me visit facility and sustained my interest in facility delivery, and I told her a lot of things, so she told me that when next she gets pregnant, she will go to the facility so as to get some gift.

Probe: What are those gifts?

Response: There is a bag, razor, lotion, pad, etc. they are much and soap.

Response: We want you to add something, so as other women will be interested in coming to deliver in the facility. First, I want you to add more medicine, add more gifts so that if a woman gave birth and gets a gift, it will help her family and her husband and other things, she will know how to meet her need, she knows that if she came to the facility she will get them. So please we want you to help us more.

Response: The reason why others will be interested to come to the facility, the truth is when I was pregnant, the nurse attendant went to my house, it makes a woman in the neighborhood that don't like attending ANC to said that she will start attending, she said it is because a nurse came to visit me with a car, she will definitely start coming, that is what makes her interested. Our own aim is that this facility (referring to the PHC in which the FGD is being conducted) is too small. When many of us came for delivery, others will be inside, and others outside because the space is too small, add more maternity ward. Add more medicine and gifts, when I go with my delivery pack bag, someone will be interested and said I use to give birth at home but I will now do it in the facility because of the bag, please add more gifts and bags so as to make others interested.

Probe: Do you think the small maternity space size is preventing others from coming?

Response: No not the maternity space, we just need more space, when we come, some of us use to start and wait outside because there is no bed. The house is not enough.

Probe: I don't understand the house, the space?

Response: This facility space.

Probe: Do you mean labor room?

Response: Yes, and water, we were here since we didn't drink any water.

Probe: let me ask you another Probe. Do you think if the facility is closer to your houses, it will make people to visit more often?

Response: Yes, it will make more people to come.

Probe: In your opinion?

Response: Yes

Probe: do you pay for delivery in the facility

Response: No, we don't pay for ANC.

Facilitator: we are going to the next session, the question we are going to talk about the acceptability of the VHW and your views about them.

Q7: How do you feel about the VHW services rendered to you? Everybody is going to tell me about what they think of VHW and their activities?

Response: My opinion about the VHW, the truth is they use to help me because when they didn't start the VHW activities, then if I am pregnant, I may stay up to 4 month without ANC but now when they start the VHW I started ANC at one month of my pregnancy.

Probe: So, are you happy with the advice they gave you?

Response: Yes, I am.

Probe: So, you think they are important

Response: Yes, they are.

Response: In my opinion, the VHW are working, they are busy with us all the time, I always pray that God to bless them, if possible, there payment should be increased because the way they are trying with us all the time. When they heard we are sick, they will quickly come and escort us to the facility. We are thankful to them and we are happy with their services.

Probe: So, you are happy with their services?

Response: Yes.

Probe: what do you feel about the VHW services?

Response: There work helps a lot.

Probe: in what way.

Response: if I am pregnant, they use to take care of me and check on me.

Probe: how do they take care of you?

Response: They took care of me when I was pregnant.

Probe: are you happy with the services they rendered?

Response 1: I am satisfied with their services.

Response 2: I am satisfy with their services, they advise us because of the advice they give to us, to be clean, remind us of next ANC appointment, because we often forget and they will refer us back to our hand card, to remind us of our ANC, so she use to really help us.

Probe: is there any other person that wants to share her view?

Response: I am happy because then I use to reach 3 month pregnancy before going for ANC, but in this pregnancy, that I just delivered, they visited me when I was one month old pregnant, I started coming for ANC, when I was sick, they quickly come and visit me.

Probe: is there any other person?

Response: We are satisfied with the VHW because since when I was pregnant, they told me about exclusive breastfeeding, and after birth, up till now they do visit me, my house and tell me more on how to take care of my baby. And what I am supposed to do the baby.

Probe: like what and what are they telling you, please elaborate more?

Response: Like they tell me after I use the toilet, I should wash my hands with soap, before taking my child and I should I should not place my child in a smoky environment, I should keep knives and razor away from my child.

Probe: did you notice any difference between this baby and the baby you delivered and home?

Response: Yes, I saw.

Probe: Is it because of the services rendered by the VHW?

Response: Yes.

Probe: What are the differences?

Response: When I gave birth to my first child, there were no serious VHW services then I was told give the infant water immediately, which I did, that baby didn’t show much growth and development like this one because this baby was exclusively breastfed.

Probe: is there any one with more explanation?

Response: I was happy with the VHWs because they use to come and advise me in my house, with my husband, they often come and advise us on how to keep myself clean, my household and even if I went to the toilet, when I came back, I should wash my hand thoroughly. That is why I am satisfied with them and they use to advise us a lot, on exclusive breastfeeding, and to avoid traditional medicine, if our children are sick, we should give quickly consult health personnel in the facility and get proper medication. And I supported my husband in this aspect.

Q8: What aspect of pregnancy and childbirth are the VHWs not helping with? Since you already told us how they have been assisting? What type of services are they not delivering, and you will like them to render, in pregnancy and delivery?

Response 1: In pregnancy and childbirth, what I will want the VHW to do for me , if she can, when my husband informed her of my labor, she should be able come, if she came, I want her to examine my stage and she will say no because she don't know how to. Her work is too conveyed to the facility. I want them to know more so as when the next I am about to deliver, she will take me check up on me and determine my stage of labor, so that we will go to the facility when it is time for delivery. We will wait together and when the stage of delivery is close, she will convey me to the facility.

Response 2: I am interested in it; I will like the VHW to check my stage of labor.

Probe: So, you want them to check your stage of labor?

Response: Yes.

Probe: before going to the hospital?

Response: Yes.

Probe: Is there anyone with a different view?

Response: What she said is correct but they didn't study childbirth so please help them with training on childbirth, because if we came to the hospital and if our delivery will be in 6 hours, we use to wait for a long period of time, with the health personnel, so if the VHW check she will be able to tell us when to go to facility. I want them to be trained so that they can help us in the community.

Probe: is there any opinion on what they can do more?

All participants' response: No.

Probe: So, all of you are in support and have no other view on how they can improve in delivering their services?

Probe: Is like you are all tired.

All participants' response: (giggles)

Response: I am in support.

Probe: Support of what?

Response: They should be employed in the facility.

Probe: Please raise your voice and explain what is your view?

Response: In my own opinion, child birth should be left for nurses because child delivery at home with the VHW use to come with complications that will require facility treatment, if a nurse is there, she will help out unlike when the baby is delivered at home.

Probe: Please add more explanation?

Response: Nurses should handle childbirth, VHW should not be involved because the nurses are more qualified and delivery at home in some cases it came with complication. If a nurse is available, she will help out.

Probe: Do you want them to be trained as nurses, so that even at home they can help out?

Response: Only in case of emergency.

Probe: how can VHW reach more women?

Response: if a woman misses her period, they use to help out, they use to visit and advise before is there ANC time, they will show her, how to work with the pregnancy in other to protect their health (record not audible).

Probe: Do you understand the question, or should we ask again?

All participants' response: No. Please repeat.

Probe: What I said is how do you think the VHW will meet other women and assist other women, like they have assisted you?

Response: In my opinion, some husband don't like the services rendered by the VHW. VHW should first advocate to the husband, then the wife because if he didn't agree the wife might not agree, that's why she should advise the husband before meeting the wife and I think that will help them.

Probe: So, in your opinion when the VHWs meet their husband, they will accept it, so u mean the husband should be contacted first?

Response: Some men do not like their women to come for ANC, so the VHW should meet the man and advise him that going to the facility is good.

Probe: How do you think other women, who have not met with the VHW, how do you think we can meet them?

Response: Women should be mobilized, married and pregnant women in an area and they should be advice in a way it will make those that are not coming to start coming.

Probe: So, you mean women should be gathered in place and be advised, is there any more explanation on how to reach more women, any other way apart from those mentioned?
Since you are quite that means the answer is no?

All participants' response: No.

Q9: Do you feel you understand the information the VHWs conveyed to you when she visits you? Do they advise you?

All participants' response: Yes.

Probe: Do you all understand?

All participants' response: Yes, we do.

Response: We understand all their explanation because when they advise us, we use to come to the facility, because of it, if not because of the information, they gave us; we would not be coming to the facility. Before we don't care about delivery in the facility, it was the VHWs, after they visited us and advise us then we came for delivery in the facility, we are satisfied with their explanation.

Probe: any other opinion?

Response: We are happy with their service, in the first instance, they use to come and advise us, because pregnancy is different some use to vomit, and body weakness, they advise us that if the signs of pregnancy are visible, it is pregnancy and we should go to the hospital. They advise us not to deliver at home and always deliver our baby in the facility. They advise us in these aspects.

Probe: is there any other explanation?

Response: They use to advise us when we are sick that we should come to the facility for health checkup.

Probe: What is the difference between the advice giving by the VHWs and the facility personnel? Is there any difference?

Response: I think they are the same because when the VHWs advice on facility visit, if we come, the hospital personnel use to complain that we came with different ill health. So, going to the facility is good and if we had used the VHW advice, we will not have encountered such issue. There work is the same.

Probe: Is there any other view?

Response: There advice is the same, because VHW advise us to come to the facility, she said if you didn't go for ANC. She will be by the hospital door to check whether you are on time or you took the right advice. That is why I said they are the same because the VHW will support you to get to the facility on time, while the facility health personnel is trying in making people to come for testing on time.

Probe: Is there any other different view?

All participants' response: (silent)

Q9: Do you understand the picture they are showing you on the flip chart?

All participants' response: We understand.

Response 1: There is a picture shown to us, those three tablets (Misoprostol) is administered to a woman after natural childbirth but not to be given to a woman that undergoes caesarean section. What is the difference? The natural childbirth was marked with good while the caesarean section childbirth was canceled.

Probe: Did the VHW explain it to you, about it use?

Response: Yes, she did but I do not understand.

Facilitator: The reason why the misoprostol was administered was to stop over bleeding which is encountered only during natural childbirth; those that underwent caesarean section don't encounter bleeding problem. So, when you deliver in the facility and you are bleeding then you will be given the miso tablet, that is the 3-table administered to you during your childbirth.

Probe: is there any other question or view?

All participants' response: (silent)

Probe: so that pictures shown by the VHW, what is the difference between the once shown in the facility, do you see them in the facility?

All participants' response: Yes, there are differences?

Probe: So, what are the differences?

Response: At the beginning of pregnancy, you will see the signs and when the pregnancy is growing.

Probe: you mean the picture is shown to you?

Response: Yes, they show me, the beginning of pregnancy and when there is problem with the pregnancy and even when I went to a facility and given birth. They will all show me and when I started to experience pain.

Probe: do you see the pictures in the facility?

Response 1: Not only the VHW showed us those pictures.

Response 2: There is.

Probe: Where is it, do you see them in the facility?

All participants' response: No, it was not pasted.

Probe: Or is there anyone that has seen them?

Response: They showed us pictures on signs of pregnancy and complications of pregnancy and effect of getting pregnant at age below 18 and above 35 and how to keep our body clean and healthy food that we can eat during pregnancy is all contained in the flip book chart.

Probe: is there any other additional information?

All participants' response: (Silent)

Q11: Do you feel free to ask VHW Probes about the information, ask them questions or do you hide other things?

Response: I ask her questions freely because she is the one visiting me, if I did not tell her my problem there is no one else to.

Probe: So, if you do not understand something, do you feel free to ask her

Response: Yes, I ask her freely.

Probe: So, you do not feel shy?

Response: I feel free because when I have a problem, I use to ask her how to go about it and she will tell me and I ask her about what I don't understand and she will tell me about what the medicine is meant for and give me advice. I asked her freely and she answers.

Probe: tell us more, do you ask freely on what you don't understand or any other question that you had?

Response: I use to be free with her.

Probe: Is there any more information?

Response: I use to ask her freely and she answers freely as I did.

Probe: What kind of questions do you ask them? If you had a question, what do you ask? Are you shy? All of you have confirmed that you are free with her, so all of you will answer this Probe; you will tell me the Probes you asked her.

Response: When I was pregnant, I asked her if my head is aching, can I take panadol or not, she said no, I should go to the hospital and get checked and if the doctor gives me medicine then I should take it.

Probe: Apart from this, is there any question you asked her?

Response: I am not in doubt about asking her, because when I was pregnant, I was sick, I asked her what am I supposed to do about my sickness, should I stop taking medicine or go to the hospital and she said to me, didn't you get those drugs from the hospital, and I said I got them she said I should take them, and I got well. And that is why every time I have issue, I will always be asked her, because I know she will help me.

Probe: Response can you please add more because earlier, you said that you have asked her Probes?

Response 1: I ask her about bleeding like washing it off during my childbirth, I use to wash off blood 3 to 4 times is that a problem? And she told me that 3 to 4 times is a

problem; I should quickly go to the hospital but if it is only once or 2 times there is no problem.

Response 2: I asked her about my paining womb and I have conducted my ANC and my womb is paining me, she said going to the hospital is not a problem (going to the hospital to address other pain apart from ANC visit) and I should quickly go to the hospital, I will get the medication that will help me and we went together and collect the medicine and get back home.

Q12: Has the VHWs house visit conducted in the community changed your perception on facility delivery?

All participants' response: No, it did not

Probe: Do you understand?

Probe: It did not change you?

All participants' response: No, they motivate us to come to the facility more.

Q13: How do you feel about the VHW as members of your community? Or are they not from your community?

Response 1: I like the idea because if she is not in my community, when I encounter health problem at night there will be no help. That is why I want her to be from my community so that if I have any problem, she will help me.

Response 2: If she is in a different area, and there is a health problem, I can't tell her to come, but if she is close, I can even knock on her door and tell her my problem and she will help me out. That is why I prefer that she is close to me.

Response 3: is good that she is close, if something happens.

Probe: You all said is a good thing, so what do you think is bad about it? Is there any problem?

Response: If she is not in our area and I started in the night, if I come to the hospital without the VHWs, the health attendant use to complain.

Probe: So now you just told me the benefit.

All participants' response: Yes

Probe: So, what are the problems encountered with them in the community.

Response: The only problem is if they are not close to us in the different community. To add more information, the VHW close to us got married in a different community, and now if she is conducting her home visit it use to take more time, she use to even reach nightfall. So please if there is a way of attaching her to a bike man to be taking her back to her house.

Probe: So, all of you are telling me that you are happy VHW stayed in your community?

All participants' response: Yes.

Probe: And there is no problem?

All participants' response: Yes.

Probe: So, no problem encountered because they are from your communities, and they know you? You never had any problem because they know you, they are aware of what is going on with your personal life?

All participants' response: No problem.

Q14: **What do you like about the VHW program? Every one of you will tell me about what she likes in the VHW program.**

Response: What I like is some of them do not know how to write and read before, but now when they stated the VHW they are able to read and write. Because when you do not know how to read there is no way you can (recording inaudible).

Probe: Did they learn how to write after they became VHWs or before?

Response: No, they do not know how to write well but now they are trying.

Probe: So, is it when they start that they learn?

Response: No, they know how to do it but not really well.

Probe: Apart from that? Response: what do you like?

Response: they helped a lot.

Probe: what type of help?

Response: They helped when we are sick.

Response: What I like about them, when they entered my house, I use to be very happy because of the health education they gave me, they use to encourage me and they use to explain a lot of things, those that we don't understand, they make me more enlighten that is why I am interested in seeing them all the time because if they entered my house, my face is lid up, and I am happy because they came, we use to gist a lot and they calm me down even when I am over thinking, you will see that my anger will disappear and we will gist. That is why I am happy about them.

Response: What I am happy about is before, nobody uses to visit us but now the VHW use to visit houses that is why I am happy with them and I want them to even get higher rank. Before there is no support, no one will tell us to go for ANC, monody will tell us to do something, some women use to even get pregnant and give birth without going for ANC, but now they have been encouraging us to go and even if we didn't they will come and ask us to go. That is what I am fascinated about the VHW activities.

Probe: xxx, what do you like about the VHW program?

Response: Even if nobody visits me in a week, they will come and visit me in my house, I like that a lot, and when they visited me, they teach me about cleanliness, and bathing my child, they told me to bath every day and brush my teeth in the morning, they told me a lot of things that are good and important, that is why I like them and

when I see them because they show me the way, how to do things, going to the hospital, eating healthy food to help me and my baby.

Probe: What of you xxx, what do you like about the VHW?

Response: What I like is if she sees me worried with my hands on my chin, she will move my hands and tell me that now is not the time to be worried. She said is not good to think much at this stage. She washed her hands with soap and water, and then picks my baby, and she asked if I have seen what she did, and I answered yes, and she said that that is what I should always do before I pick my baby.

Probe: So, they asked you to be clean.

All participants' response: Yes

Probe: So, they advise you on how to be clean, go for ANC in the facility and gist with you?

All participants' response: Yes.

Probe: How many minutes do they spent per visit?

All participants' response: About five minutes.

Response: What I like is they met me when I was cooking and they waited until I was done, they are patient with me. She talks to me on cleanliness to prevent me and my child from contracting disease, advise us on how to stay healthy, she check my ANC card.

Probe: is there any further opinion?

All participants' response: (Silent)

Q15: What don't you like about the VHW program?

All participants' response: (Silent)

Probe: What is it that you don't like, I know you all told me the aspect of their program that you liked, so now tell me what you don't like?

All participants' response: Nothing.

Response: I always like them and what they do.

Probe: How many times do they visit you in a week?

All participants' response: Twice.

Probe: Twice a week? Do they come every week?

All participants' response: Yes.

Probe: Is there any more information or comment on issues that we didn't discuss or asked? That you feel is important?

Response 1: What is happening now has enlighten me, before I don't give birth in the facility, I gave the baby traditional medicine, but now that I am enlighten, I am doing exclusive breastfeeding.

Response 2: When I gave some of my older children traditional medicine (when they were infants) they got sick a lot but this girl I am only breastfeeding her, I don't give her water and she don't fall sick a lot. They have enlightened me and I am now aware of the difference between what I practice before and now this girl, I didn't even encounter any problem. Even when she is teething but with the previous kids, I really suffered, there was a particular wound (infection) on the child's head and we normally block it, and it will result in swelling of the child's head. I did not know it was a problem.

Facilitator: Thank you, is there any more information on facility visit, your village health workers, or questions?

All participants' response: No.

Facilitator: Now we have come to the end of our FGD, we thank you all, so much for your help and contributions, we apologies for keeping waiting and we thank you for sharing your views. I pray you all reach your destination well and safely. Amin

Probes Views

1. Almost all the participants are actively participating but their voices are sometimes inaudible. This is coupled with their children crying/noise in the background.
2. xxx: was the most active participant but not dominating.
3. Sometimes the all the participants give a unanimous respond hence the respondent name in the transcribed is tagged as participants.
4. xxx: was quite shy hence the facilitator took notice and engaged her in conversation.
5. Facility, hospital and health facility are used interchangeable in the transcript.

End of Recording

Focus Group 2

TITLE: Focus Group Discussion with women who delivered in the facility within the last 12 months.

NUMBER OF PARTICIPANTS: 11
DURATION OF RECORDING: 1hr 2mins.
Definition of terms
ANC - Antenatal care
PNC- Postnatal care
KMC- Kangaroo mother care
ETS -Emergency Transport Scheme
BP- Blood Pressure

[Beginning of recorded materials]

Access to facility delivery
Facilitator: Let's talk about getting to the health facility.
Q1: First, tell us a bit about how you travel to the health facilities. Probes: are the facilities too far from where you live or hard to get to? Do you have to pay transportation?

> **Response:** when I started labour at home there is an emergency driver (Emergency Transport Scheme) who convey me to the health facility, and I was received by the facility health providers.

Probe: you mean you used the ETS during delivery?

> **Response 1:** Yes
> **Response 2:** When I started labour, I used motorcycle to go to the facility

Probe: All participants used ETS except for one participant, who used motorcycle?
Probe: Do you pay any money to the ETS driver?

> **Response:** ETS It is free, we don't pay.

Q2: How does your husband feel about you delivering your baby in the facility? Probes: why do you think he supports you to deliver in the facility? Why do you think he doesn't support you deliver in the facility?

> **Response 1:** My husband always supports me to deliver in the health facility, he knows that whenever I deliver in the facility is will be taken care of, I will be healthy and my baby too.
> **Response 2:** My husband supports me, because he wants me and the child am carrying to be healthy. He loves that I deliver in the facility.
> **Response 3:** The reason why my husband or my daddy supports me to deliver at the health facility is because in the facility the healthworkers will check me, test me and also check the position of my baby. He also asks the healthcare workers about my health.
> **Response 4:** My husband is always happy, and he supports me because of the free drugs I will be given, and the drugs will prevent me from diseases. That's why he supports me.

Q2: How does your mother in law feel about you delivering in the facility? Probes: why do you think she supports you to deliver in the facility? Why do you think she does not support you deliver in the facility?

Response 1: My mother in law is happy. Before they stop us from going to the facility for delivery, but now everything has changed because of awareness, she always insist I should go for ANC and deliver at the facility because she said during their time they really suffered but now everything has changed.

Response 2: The reason why she's always concerned about my facility delivery is for me to receive care, check-up, drugs, and for me and my baby to be healthy, she said they suffered during their time.

Response 3: Our mother in-laws support us because there are complications during childbirth like excessive bleeding, convulsion, so when you deliver in the facility even if you have such complications you will be treated.

Probe: you mean your mother in laws support you all to deliver at health facility?

All participants' response: yes, they support us and they are happy.

Response 1: They are now wise

Facilitator: Now we are going to our next section which is: Views and Experiences about facility deliveries. let's talk about the care you get when you come to deliver at the health facility**.**

Q3: How did you feel when you come to the facility to deliver your baby? If you have never delivered in the health facility, tell us the experiences of other Probes: what usually happens - are you seen right away, or do you have to wait long? Why is that the case? What do you we should do to improve this? Do you feel that through the staff treated you with respect and value your point of view?

Response 1: When I came to the facility when I was brought to the facility, they check my BP, and the position of my baby.

Response 2: when I was brought to the facility when I was in labour, when I came in the nurse that received me, received me with a smile and laughter and asked me what is the problem? I told her I was in labour. When I told her I was in labour, she placed me on the bed, she examined my abdomen, when she examined my abdomen, she checked my blood pressure, and kept me on the bed to rest.

Response 3: When I started labour, I was taken to the facility, on reaching there the nurse smile at me and ask me what's wrong with me, I told her her it's labour. Then she laid me on bed and measures the pregnancy and my blood pressure. Then she asked me to rest on the bed.

Response 4: I was taken to the health facility when my labour started, immediately I arrived at the facility premises, the nurse who assisted me asked, are you OK? I am I answered here by telling her that i am in pains, she checked me and confirmed that it was really labour. then laid me on bed and check my BP. She also checks to be sure it's labour, she confirmed it and said it is. She asked me to relax. I am happy because she took good care of me till I delivered.

Response 5: When I started labour, I was taken to the health facility. The health worker who assisted during my delivery, checked me, and confirmed that I was about to deliver. I was taken to the bed where I laid on. They kept checking on me regularly till I delivered my baby. took good care of me and ensured I am healthy and the baby as well.

Response 6: I was taken to the health facility when I started labour, the health worker that received me, was with me throughout the process and kept checking on me at regular intervals till I delivered safely. She took very good care of me. and the facility health worker took good care of me she was with me throughout till I delivered, and I delivered successfully.
Response 7: when I was taken to the facility, the workers were around and one of them ushered me into the facility and me what was wrong. I told her that I was bleeding, so she said I should come, she checked me, and said it was really labour. She took good care of me till I delivered and went back home.
Response 8: When I started labour I was taken to the health facility, the health workers said I should lay on bed and she checked me to ensure am due for delivery, then she assisted with the delivery and after the delivery I started bleeding, then they checked my abdomen to confirm there is no other child in there, then they gave me 3 tablets to take, and that's all.

Probe: Apart from the 3 tablets did the health worker give you anything?
Response: No

Probe: So, you mean all of you received care from the facility during your delivery?
All participants' response: yes

Probe: Did any of you experience any problem or do you have to wait to for long before been attend to?

All participants' response: No Problem and we don't have to wait; we are seen right away.

Q4: How is delivering at the facility different from delivering at home? Probe: compare the services you get from the TBA to services you received from health care providers at the facility.
All participants' response: Yes, there is difference between facility and home delivery.

Probe: I want to know the difference, everyone will talk
Response 1: There are differences between facility and home delivery. when a woman delivers at home with complications like bleeding, there is no treatment available. The three tablets that are given to us at the facility are not available at home. There are challenges with home deliveries but facility deliveries you get optimum health, but home delivery is not good; you will not get optimum health.
Response 2: There is difference between home and facility delivery. At home there is no care. In the facility, healthcare workers take good care of you very well. When you deliver to home and start to bleed, you could die at home, but if you deliver at the facility, the health workers will know how to treat you. In the facility, there is postnatal care but at home nobody will give you that care, sometimes when you had excessive bleeding and if it is home delivery, a woman might lose her life, but if it is facility delivery she will be taken care of.
Response 3: There is difference between home and facility delivery. In the facility when we come, they take care of us. after delivery, the health workers will check our abdomen and expel bad blood. They can give us medication or injections for

excessive bleeding. They can give us injections we and the baby, but at home there are no injections available for us or for the baby.

Response 4: After delivery in the health facility, and the umbilical cord has been cut, we will be asked to place our babies on our chest to feel the warmness of the mother (KMC), they will clean the baby thoroughly, rub oil on him, Wear him cloth clean the umbilical cord, and by so doing it will prevent the baby from any form of illness that will affect the umbilical cord., and I am very happy when i gave birth. While at home nobody will do that to you or your baby.

Probe: you said they normally clean the cord and applied oil to it what do they applied to the cord if it is home delivery?

Response 1: Nothing

Response 2: for home delivery, when I delivered at home, when I started labour, I was taken to facility, and we waited I did not deliver so I was brought back home. But when I delivered at home, after delivery, I started bleeding excessively, they poured hot water on me, then I fell, and I was taken back to the facility. Later, understood the difference between facility and home delivery, my later deliveries at the facility, since when I a on the bed, they gave be injections before I came down. So, I realized there is difference between facility and home delivery, and I enjoyed facility delivery better than home delivery.

Probe: The home delivery, which baby was that is it the current baby

Response: no that was my first baby

Probe: do you acknowledge that facility delivery is better than home deliver?

All participants' response: yes

Response: When I gave birth to my first child at home I had complication which was heavy bleeding, and the only thing they did to me at home was to pour hot water on me, then I fell down and I was taken to the health facility, which I was treated at the facility. Ever since then I came to realise the difference between home and facility delivery.

Q4: Compare the services you get from the TBA to the services you received from a health provider at the facility.

Response 1:The difference between the services we get from TBA with that of facility health providers is that, when the TBA comes to us, she advises us to go to the facility for delivery that she will not assist us in delivery. She advises that when we are in labour, we should come to her so she can escort us to the facility. While healthcare workers in the facility when we come to them they will check us and if it is real labour, when we deliver the baby, they cut the umbilical cord and place the baby on the mother's chest, after 24 hours, the brain will know who the mother of the child is so that the breast milk will be stimulated. Then the baby will be put to the mother's breast and start sucking. That is the difference between the skills of the TBA and healthcare workers in the facility.

Response 2: The difference is that the TBAs only functions at home even if I gave birth at home, she (TBA) cannot give me care at home that can be compared with that of facility, she cannot inject me, she probably does not have the skills to inject me. Even if she does, it is not her work. Her work is just to help with the delivery. But

in the facility, you get the best care. When I deliver in the facility, I get medication, injections…..

Probe: is there any other differences apart from the ones you make mention?

Response: no

Q5: Do you have any special religious or cultural requirements - such as needing to be seen only be a female health provider? Probe: overall have you found the staff to be welcoming?

All participants' response: no cultural or religious requirements

Response 1: I would prefer a woman to assist me during delivery because if am naked I don't have anything to hide to my fellow woman, but I will be shy of a man.

Response 2:The reason why I prefer a woman to assist me because she knows the pains in childbearing, so even if I am pain, I might cry or stan up, she will not be angry because she understands the pain I am going through.

Response 3: I prefer a woman to a man, because a man is not sympathetic. A woman is sympathetic, and she knows the pain of labour, but man does ….

Response 4: I prefer a woman. Like me if a woman see me struggling with labour, she can rub or massage me which will ease my pains but a man will not do that because he has no idea or understanding of the kind of pain I am going through. But a woman knows my pain she will console me and laugh with me

Response 5: the reason why I prefer a female to take my delivery, is because a female when in labour, you are naked, no clothes male will be too shy to be looking at you and touching you but female will touch you freely and check you till u deliver

Probe: you all prefer female health workers?

All participants' response: yes

Q6: What do you think can be done to facilitate you deliver in the health facility? Probes: ease access; improve quality of services, user fees.

Response 1: If the facility is close to us everyone will have easy access to it and will deliver in it.

All participants' response: The services are free; we don't pay money so it should continue that way.

Q7: What do you think can be done to facilitate other women to deliver at the health facility?

Response 1: The best ways is to advise them and tell them the importance of facilities delivery. By telling them that home delivery might result to complications and one will not be given tablets or injections at home.

Response 2: By telling them that they do not need to pay any amount for the services, it's free. By telling them this they would like to come.

Response 3: By giving incentives (gifts) to women who delivers at the health facility will make other women come to the facility during delivery.

Response 4: Back then people usually deliver at home and because of the complications they experience they later stop delivery at home but the health facility. We advise them that facility delivery they will get care.

Probe: what advice do you think can be given to those that do not go for facility delivery?

Response: By advising and telling them the type of care we received from the facility if they deliver at home there is no care, no injection, no drugs, nothing but if it's in the facility it will be different.

Probe: Do you think by you advising them will make them come to the facility?

Response 1: If I talk to the women and they refuse I will talk to the VHW if she agrees to tell the village head about the women, then the village head will talk to them directly.

All participants' response: By telling the village head to talk to the women directly.

Probe: Do you think the women will change if the village head talk to them?

All participants' response: yes, they will

Response 1: in my community the village head said any woman that refuse to go to the facility should be reported to him.

Facilitator: The next part of the focus group looks the views and Experiences of participants with the VHW program. Let us talk about your experiences with the VHWs in your area.

Q7: How do you feel about the VHWs? Probes: How do you feel about the services they provide to you and other women?

All participants' response: They are doing very well. We are happy about everything they are doing.

Response 1: The VHWs are doing their best by advising us. When I was pregnant the VHW visited me and told me to go for Antenatal care in the health facility, she told me that I will receive a lot of care. She also said I should start it when the pregnancy is 3-4 months old. She also talks to me about personal hygiene.

Response 2: What the VHWs are doing is particularly good. They advise they give us and many more.

Response 3: What they are doing to us, to be honest they are trying, for instance she always come to my house to advise me, and she visit me like 3-4 times a week and she's really doing well.

Response 4: Whenever the VHW visits me she advise me on personal hygiene and I use to tell her that I wish the government will employ her and I will also benefit from her.

All participants' response: we feel very happy with their service.

Q8: What aspect of pregnancy and childbirth are the VHW not helping you with, that you would like them to help you with?

All participants' response: None

Probe: How can the VHW reach more women like you?

Response 1: There are areas that are lacking VHWs, so if it's possible they should also be given VHWs so that they too can benefit the way we are benefiting. It will also reduce the rate of home deliveries in such communities and also cases of woman with premature babies will be reduced.

Response 2: Most especially we in rural areas, we have Fulani around us who doesn't have VHWs, so if it is possible, the Fulani women should also be trained as VHW. It will also reduce issue of home delivery in their communities.

Probe: Do you mean they should also recruit and train the Fulani women to become VHW.

Response 1: Yes

All participants' response: All our contributions are the same

Response 2: Mine is for those that are in distance communities the VHWs cannot reach them; if there is money their transportation should be paid.

Q9: Do you feel you understand the information the VHW conveys to you when she visits you? Probes: How is the information she conveys to you different from the information you get from the providers in the health facility.

All participants' response: yes, we all understand the information.

All participants' response: The information is always in line, it's the same.

Response: the difference between the information the VHW conveys and that of health providers is that, the VHW advices me and always visits me at home to ensure I practice it while the health providers only advise us in the facility.

Q10: Do you feel you understand the flip chart the VHW uses for health education? Probes: do you see these kinds of charts in the facility? How are the charts in the health facility different from the ones you see with the VHWs?

All participants' response: Yes, we all understand the flip charts.

Q11: Do you feel free to ask VHW Probes about information she delivers to you? Probes: what kind of Probes can you ask her? What kind of Probes are you not able to ask her?

All participants' response: yes, we always feel free to ask her anything we are not clear with.

All participants' response: yes, we feel free to ask them Probes about information they deliver to us.

Response 1: I once hide something from her. When I had vaginally itches and whitish discharge during pregnancy, I was shy to tell her whenever when she asked me if I have any problem, I later told her everything and she told me to go to the facility which I did and I was treated.

Probe: What is the kind of questions do you ask VHW?

Response 1: Ask her about the solution to my bleeding during pregnancy, she told me to go to the facility to get medication, I did, and I was cured.

Response 2: I asked her about the causes of premature babies, and I compared her answer with the one I was told at the facility, which was same.

Q12: Has VHW visits to the community changed your perception of facilities deliveries?

All participants' response: it does very well.

Response 1: Back then we only go to the health facilities 3-4 times for ANC but now our perception has changed, we go for ANC, Delivery and PNC.

All participants' response: we have same Response

Q 13: How do you feel about the fact that VHWs are members of your community? Probes what problems do you think is associated with this? Why is that a good thing?

All participants' response: yes, we are happy that they are our community members and they always visit us.

Q 14: What do you like about the VHW program?

Response 1: What I like with the VHW is during my pregnancy, she usually visits me early morning to remind me of going for ANC and accompany me to the health facility for ANC.

Response 2: she sometimes helps me with my chores (washing and sweeping) especially the difficult ones.

Response 3: Back then my mother usually helps me but now the VHW have taken her place because they always help me.

Response 4: The VHW advices me and sometimes bring fruits for me

Response 5: the way the VHWs are taking good care of us during pregnancy and after delivery.

Response 6: I love their bags and the way they always hold it in feel like becoming a VHW too.

Response 7: I like the way they always advise us.

Q15: what do you not like about the VHW program?

Response 1: Whenever a VHW forget to greet me I call her back and tell her why she didn't greet me.

Response 2: sometimes they tend to take much of my time and it makes me angry but it's not all the time.

Facilitator: We have come to the end of our discussion; I don't know if you have anything to tell us or questions.

Participants comments

1. My name is xxx my questions goes like this, we earlier said some of the communities are lacking VHWs, I want you to consider those communities by training more VHWs to be helping those people.
2. My name is xxx. This VHWs program is it sponsored by government or company?
3. Facilitator: this VHWs are women who volunteered to work for their communities in other to help other women, so society for family health with collaboration with the government train them to become VHWs. They started this program without being paid but now they are receiving small stipend.
4. Again xxx, these ETS drivers whenever their motorcycle is faulty can the organisation help them? For example, if during labour and there are 2 women at the same time, how can they convey them?
5. Facilitator: there are ETS drivers in most of the communities. He can convey one person and come back and pick the other. And the ETS are in the communities where there are facilities, that's why you all have been given their numbers, all of you here said it is the ETS that help you during your deliveries so u see they are really helping. I believe none of you have any difficulty in contacting them and none of them have ever refuse to carry you to the facility. But we are hoping that you will get more ETS drivers.

Response: is there a reason why the ETS drivers are not being paid?

Facilitator: Just the way we all stated that they are doing this work to help you and your children, just the way they train the VHW that's the same way they train them, and all the activities they are doing is voluntary and anyone that says he wants to volunteer will never expect anything from anyone. But if you feel you can give them money to fuel their vehicles no problem you can do that but they said this work they are doing is volunteer and they are doing it to help their communities.

Facilitator: are there any contributions? If none thank you for your time we are grateful.

Views

1. Time management was effective.
2. All participants are open to sharing their point of views.

Focus Group 3

TITLE: Focus Group Discussion with Mothers Who Delivered at the Facility
DURATION OF RECORDING: 40 min, 20 secs

Number of Participants: 10

[Beginning of recorded material]
Q1: How you travel to the health facilities?

Response 1: when I want to come to the facility, I ask permission and I walk to the facility because I live close to the facility.
Response 2: When I am ready to come to the facility after asking for permission, I walk because I am close to the facility for antenatal but when I was coming for child birth, I was brought in a car by my husband who is also an ETS driver
Response 3: I also walk to the hospital for ANC because I am close to the facility. I live in the same area with Response (part.1). When I was coming for delivery, I was brought with a motorcycle
Response 4: I also walk to the facility because I stay in town. When I was coming for delivery, I was brought in a car, motorcycle by my husband.
Response 5: I also walk to the facility for ANC. Even when I was coming for delivery, I still had some strength left; I came to the facility on my feet.
Another woman also goes walks to the clinic because she is very close to the facility; share the same wall will the facility
Response 6: I come with a car

Q2: How does your husband feel about delivering your baby at the facility?

Response 1: Honestly, most of our husbands are in support of it
Response 2: our husbands are in support of facility delivery because of the care we get in the facility

Q2: How does your mother in-law feel about delivering your baby at the facility?
All participants' response: no problem

Response 1: there is no problem sometimes they do not even know you came to the facility to deliver
Response 2: Except you are living in the same house with them, they will not even know about it. We do not live in the same house with them/her. Even before the advent of VHWs we have never had problems with our mother in-laws in terms of going to the facility to deliver.
PROBE: why do you think they are supportive of you delivering in the facility?
Response 3: they agree because they know the importance of facility delivery. When the VHW come for sensitization, they engage all of us like 10 women at the sometime, including the mother in-laws so; they don't have any problem as regards to that. But even before the VHWs started working, our mother in-laws were supportive of facility deliveries.
Response 4: even though they (the mother in-laws) have had their babies at home,
Response 5: you know childbirth is not as it used to be in the past. They do not want their daughter in-laws to deliver at home. There are lots of complications and people have to use the facilities.

Q3: What kind of care did you receive when you delivered at the facility?

Response 1: They try a lot. They take care of use when we come. For instance, cases of protracted labour, they keep checking on us to tell us the expected delivery time like so hour and so. When they tell us the delivery time, we inform our people at home to bring the things we will need for delivery.

Probe: you do not come with baby clothes when you come to deliver?

Response 1: we do not come with baby clothes; we just come until the healthcare providers check us and say that it is real labour…then we inform our realities

Response 2: There is false labour, sometimes even hot fever could be perceived as labour but when you come to them and they prescribe medication for you, and you take that, the fever will be taken care of.

Response 3: only when the healthcare workers say it is delivery time…

Response 4: sometimes, there are pregnancy labour that might not be actual delivery labour so, when we come, if it is ascertained that the baby is coming then we will bring our things for delivery.

Response 5: For some women, they will be able to know from home because since she has given birth more than once or twice. She will know if it is labour and she will bring her things with her from home so that there won't be any need to go back home for the things.

Response 6: we don't wait for long. Immediately you arrive, they will attend to you, if you come for delivery, they will accept you immediately

Response 7: once you come for delivery and the healthcare workers are informed, they will even come running to attend to you. Even if they are in their homes and they hear a car stop, and they are informed, they will even run to the facility.

Response 8: The workers are really trying. Even it is at night, they come quickly to attend to us.

Q4: What is the difference between facility birth and home delivery?

Response: I don't know the difference because I've never given birth at home.

There is difference. Once you are in labour you leave your house, you are coming from the house with all the things you will need for the delivery you come to the facility, once you get there and they check you and confirm that it is real labour, they pay very close attention to you they will never leave you by yourself they will be with you till you deliver.

Probe: what I am asking is what is difference between the care you get in the facility and the care you get when you deliver at home?

Response 1: I have never given birth at home

Response 2: I have experience with home birth: there is a lot of difference; the facility delivery is much better. Since I have never given birth at home, I decided to try home delivery with my last child, let me try. You know at home; some will sit down on a chair or hold on to a chair to deliver. In the facility you will be given a bed you will be given a bed. I decided to give home birth a try, honestly, health facility birth is better than home delivery.

Probe: is the bed the only attraction to facility delivery?

Response 1: no, the care received at the facility is better compared to that received at home.

Probe: when you delivered at home did you have any help? You said there was no TBA

Response 1: no TBA, it was just people that were with me at that time that helped me.

Response 2: I have never had a home delivery, but there is a lot of difference between home and facility delivery. the care received at the health facility is better than that received at home. In the facility they will squeeze your stomach to make sure they control the bleeding. Sometimes at home, when you deliver, the placenta will not fall, so they will shake you, do this and that, to try and get the placenta to drop., but in the facility once you give birth, they will manually remove the placenta and do all that is required. At home they will give you home remedies, which some to be coughing, it might even make you anaemic, which might result in them having to take you to a specialist hospital for surgery to take out the placenta. But when you deliver in the facility, they will refer you to a specialist hospital immediately they sense a complication they cannot handle. Facility deliver is better.

Probe: that means no one of you likes home delivery

Response: none

Q5: Do you prefer male or female health worker to attend to you during childbirth?

Response 1: I prefer a female because she is a woman like you and would understand the pain you are going through more.

All participants' response: we prefer females

Response 2: you know we Fulanis are very shy. I prefer a female but if there is no female personnel on ground, you will have no choice because while in labour pains, you will not care whether it is male or female. Your only concern would be to give birth and rest. I have had a man attend to me when I delivered onetime in the past. But my other deliveries, I had female healthcare providers attend to me.

Response 3: we prefer female healthcare providers to attend to us for delivery. When you come to deliver, and they must examine you to know the progress of your labour, it will be better of the provider is female. Better than male because with a female you feel freer to express everything to her; you will not hide anything from her so it's better.

All participants' response: we all prefer female healthcare workers

Q6: what do you think can be done to facilitate your/other women delivery in a health facility?

Response 1: you see when you deliver in the facility, they have taken care of you kept your body clean, including your baby, when you return home, you will meet a clean environment. But if you deliver at home. if you give birth in the facility, that medication they give, misoprostol when given to you it will arrest post-partum haemorrhage unlike the woman who delivered at home.

Probe: I understand, but the Probe is what will attract you to deliver in the facility?

Response 2: what will further entice us to come and deliver in the facility, is when we deliver, we get good care, our health and our baby's health is taken care of, and you return home healthy.

Probe: do you not get that kind of service from the facilities now?

Response 3: we do get those kinds of services now. And they give us soap, pad, and chlorhexidine in a bag

Probe: apart from that what else do you think will entice you to deliver in the facility, even for other women that do not want to deliver in the facility?

Response: In the past, the kind of medicine they give cholorhexideine, and that bag that they now give, they did not use to give women before. Now they give women who come to the facility to deliver, those items. When another woman sees those gifts; she will say, I will also go to the facility to deliver so that I can also get this type of bag. In the process, you realise that you are influencing a lot of women to deliver in the facility.

Probe: so, meaning it is those gifts that will entice women to deliver in the facility

Response: yes sure

Probe: do you pay do deliver in the facility?

All participants' response: no, it is free of charge

Response: you only must pay if you develop a complication and an extra drug has to be used on you. You will have to pay for that extra medication. Because like my other son, when I came, I delivered normal with no complication, so I did not have to pay any fees.

Probe: when you have a complication during delivery, and you are asked to pay extra fees, how much are you usually required to pay?

Response 1: there is a delivery I had her, they induced my labour with a drip; at that time, misoprostol was not free of charge, you have to buy for yourself. So, we had to buy

Probe: how much did you have to pay?

Response 2: I do not know; I was in labour at that time. But not it is given at no cost.

Q7: How do you feel about the VHWs?

Response 1: We are really enjoying how they do their work. As early as when the pregnancy is three or four months old, they come to tell us to go for antenatal, because of the fever can affect the baby's health. They advise us to go for 4 ANC visits. They keep checking on us, may times they come to visit us and tell us to go to the facility on time so that your heath and your baby's health will be cared for.

Response 2: They also tell us to use mosquito nets to protect ourselves from malaria…unintelligible

Response 3: Some women don't even have the intention of visiting the facility for ANC, but because of their persistent visits, they now go and when we go, we enjoy the facility visits because those drugs that we are given assists us a lot and protects us from diseases and make us healthy.

Response 4: They tell us to eat good food for the sake of our health and that of our babies

Response 5: When they come for visits, they tell us to eat good food that will keep us healthy like eggs, liver, yam, bread, just good food that will make us healthy. That is why we enjoy their visits into our homes.

Response 6: we enjoy their visits

Q8: What aspect of pregnancy and childbirth would you like them to further assist you with?

All participants' response: there are none

Probe: what would you like the VHWs to do for you that they are currently not doing?
Probe: anything you want the VHWs to do for you that they are currently not doing?

Response: There is none

Probe: How do you think more women like you can be reached by VHW?

Response: They can go to their community ward head and tell him that we have been hearing about the VHWS but they have not reached us and the community head can do what is within his power to connect the women with the VHWS.

Q9: Do you feel you understand the VHW conveys to you when they visit you?

All participants' response: We understand everything

Q10: Do you feel you understand the flip chart the VHW uses for health education?

All participants' response: yes, we understand the pictures very well

Probe: what is difference between the pictures the VHW show you and the ones you see in the facility?

All participants' response: No difference

Q11: Do you feel free to ask the VHWS questions about the information she conveys to you when she visits you?

Response: We feel free with them because while we live in the same community with some, we live in the same house with others.

Probe: What questions do the VHW ask you that you find difficult to answer?

All participants' response: None

Q12: Has VHW visits in the community changed your perception of facility delivery?

Response 1: In the past, women do not come for ANC till they are almost due before they come and register for ANC at the facility. But now, women with start registering for ANC when their pregnancy is only about 3-or 4-months old pregnancy will start coming for ANC visits because of the VHWs sensitisation.

Response 2: You know some women don't want to go to health facility completely but due to the information provided by VHWS, they now go.

Q13: Are you happy that the VHW is a member of your community?

Probe: do you encounter any problems because VHWs are part of your community?

Response: no problem at all

Probe: do you prefer that the VHW is from your community?

All participants' response: yes we like that

Response 1: If the VHW is from your community, you will feel freer with her and you will not hide anything from her. But if they are from different communities, you will not feel free with them. The VHW as well, will also tell you everything if you are from the same community and you interact. We prefer women from our immediate communities.

Response 2: Honestly, we feel our community members are better. We will not refuse if they are from other communities but those from our communities are better.

Q14: What do you like about the VHW programme?

Response 1: What I like about the VHWs is they go around the community and register us for ANC and encourage us to go for ANC appointments on time. If you do not go for your appointment, they will re-visit you and encourage you to go for your ANC appointment at the facility. Even some women that had no intention of going for ANC, will eventually go. When they meet your husband at home, they will ask him why you have not gone for your ANC appointment? So they emphasize on the ANC appointments.

Response 2: I like their regular visits to our homes.

Response 3: more than everything they sensitize women about the importance of going to the facility and keep reminding us of our antenatal visit days. I enjoy their visitations a lot. They give us medication, so before you start going to the facility for ANC, you can start taking the medication they have already brought to you. So, you see that is protective before you come to the facility for further check-up. I like the fact that they go around the community.

Response 4: I like the fact that they sit among us and explain things to us. I really like that.

Response 5: If they come, they tell us about the sort of good food (health options) we are supposed to eat

Q17: What don't like about the VHW?

Response: we like everything about them

Probe: would you like to be a VHW?

Response 1: yes, I would love to

Response 2: There is nothing I don't like about the VHW

Response 3: nothing

End of Recording

Focus Group 4

TITLE: Focus Group Discussion with mothers who delivered their babies at home

DURATION OF RECORDING: 53 mins 32 secs
NUMBER OF PARTICIPANTS: 10

[Beginning of recorded material]

Q1: Let's talk about how you get to facility?

Response 1: I walk to the facility because I reside near the health facility.
Response 2: I walk to the facility during ANC but utilize car or husband motorcycle during delivery

Probe: have you ever utilize emergency transport drivers before, do you know this driver?

All participants' response: …….yes we know and utilize them
All participants' response: yes, we use ETS
Response 1: I go well I walk to the facility even for delivery also because I stay close to the facility.
Response 2: I also walk to the facility
Response 3: I walk to the facility because I stay close by
Response 4: we utilise emergency transport drivers to take us to the facility for delivery

Probe: But you delivered at home

All participants' response: We delivered at home

Probe: you delivered all your children at home?

Response : yes but when you deliver at home, the placenta does not come out at home, so you get ETS to the facility to get the placenta out. When you called them they responds even if one deliver at home where one have delay placenta, you can utilise this drivers to convey you to the facility to have this placenta removed

Q2-How does your husband feel about delivering your baby in the facility?

Response 1: they cooperate with us unless there is an issue. He always allowed me to access services at the facility because of the care one received at the facility. I delivered at home because of health workers strike, before I could get a car to access facility service elsewhere, I delivered at home.
Response 2: Delivery at facility and ANC at facility is better than home delivery. Before I could get car to access facility services I delivered at home, if not I would not have delivered at home. when I delivered at home, I came to the facility because of retain placenta

Probe: xxx, this is your first child?

Response 1: yes
Response 2: He support me 100% to access facility services because of care and advice on what to eat and where to lay during your pregnancy period in the facility.

Response 3: He does not have issue with me delivering at facility. He was not at home during my labour he could have rushed me to the facility immediately. He only heard of my delivery at home

Q2-How does your mother in law feel about you delivering your baby in the facility?

Response 1: she allows me to for ANC and delivery. She would not even allow me to stay at home without coming for ANC and delivery. She always allows me because of the importance for healthcare and to avoid complications. She ensures I attend ANC and when in labour she calls her son to convey me to the facility immediately without delay. She is incredibly supportive.

Response 2: she cooperates with me to go to the facility she fights me when I fail to access facility services because she wants me to always be in healthy state. We visit facility to stay healthy

Response 3: They agree and cooperate with me to access facility services. Delivering at home come with complication because when one start bleeding at home, they will not know what to do. And one would have been exhausted before getting to the facility. you cannot access care available in the facility. The mother in law will become confuse when they notice bleeding or complications. One gets tired before going to the facility that is why they just one me to deliver in the facility.

Response 4: she allows me access facility service because of fear and to prevent complication associated with labour and delivery.

Response 5: she wants to always prevent problem with delivery at home that's why she allows me access facility services. I deliver at home because facility staff are on strike at that time

Response 6: for my health and that of the unborn child is the reason she allows me access facility services. They want me to immediately access services at the facility

Response 7: she fears complication associated with home delivery for bleeding and retain placenta which might lead to loss of life this among reasons she allow me come to the facility for delivery.

Q3- How did you feel when came to the facility to deliver your baby? Are you seen right away?

Response 1: I was attended to immediately there is difference between facility care and care at home. At facility they use drugs that stops bleeding, helps in resolving retained placenta issues and drugs to dry the naval of the newborn, they clean up the child immediately after delivery but this is not the practice at home.

Response 2: I was attended to immediately .when I gave birth, the in- charge clean and inject me and baby and she gives me drugs to take, she had great respect for me and we went back home safely.

Response 3: They take good care of me. Immediately I arrived I was received, and they begin their examinations. They do everything for you until you deliver safely.

Response 4: They are always around even at night to attend to emergencies

Response 5………………………..

Q4-difference between facility delivery and Home delivery

Response 1: I am not talking about this facility but the facility I went to I was not attend to on time because of queue in the facility. Before they can attend to me, the baby is already coming out. Without queue facility delivery is better because Facility staff takes care of you more than TBA. Because they do not things traditional like the TBA. Facility staff inject and give you drugs to help you and one cannot get that at

home. I have given birth at home and facility and I prefer giving birth at facility, but my delivery always happen before I can access facility services

Response 2: one will not be expose to disease in the facility compared to delivering at home and again the facility staff are more experience and gives more attention and care than the TBA.

Response 3: There is a huge difference, delivering at home, TBA will not know when their complications they only wait for a child to receive but facility staff examine and tackle complications. They do check up

Response 4: There is big difference between facility and home delivery. Facility can tackle complications during labour legs or hand can come out, in the facility they can handle this but TBA cannot handle complication rather they become confuse, they begin to spit and hold rage.

Response 5:There is a huge difference, home delivery comes with a lot of complications The care one gets in the facility cannot be compared to the one at home and in case of complication of swollen body or bleeding this can be control at facility and not at home. If this happen at home, you will be rushed to the hospital.

Response 6: delivering at facility is far more than delivering at home because when there is complication at home you will still be rushed to the facility.

Q5- Do you have any special religious or cultural requirement such as needing to be seen only by female healthcare provider? How has the staff responded to these needs?

All participants' response: Female

Response 1: I prefer female. only women can handle women issues. Because female understand you.

Response 2: I prefer female health worker because we share same gender. I relate freely with a female health worker but when there need for a male health worker to help, I do not care.

Response 3: I will prefer a female health worker to attend to me because I will have this belief she knows what I'm going through and know how to relate with me better than a male health worker who has not witness labour before.

Q6-What to do you think can be done to facilitate you and other women to deliver in the health facility?

Probe: what will be done that will attract you and other women to facility to deliver

Response 1: that will improve our health

Response 2: in some facility, the money paid is extremely high, so reduction in service charge and free service and incentives given freely will attract women to facility to deliver.

Response 3: like she said free services will attract women to facility to deliver and free drugs and soap also attract, free blankets

Response 4: Like they said, incentives like caps, bags socks will attract women to deliver at home. Sometimes if you deliver at facility and this incentives are available you will be given but when they are finished they let you know is finished and encourage you that next time you deliver you will be given

Probe: so, you want this incentive to be increased?

Response 1: yes, sometimes you get, other times you do not. But when is always available it will trigger facility delivery?

Q7- How do you feel about VHW services they provide to you and other women?

Response 1: I am enjoying their services. Sometimes even if you are reluctant in access facility services but after their visit, I just change my mind to avoid problem and visit the facility. Sometimes even if the husband has issue with you attending facility the VHW have away to convincing him into allowing you attend facility.

Response 2: the way she said is true, we are enjoying their visit

. The VHW has a way of persuading one to attend facility by the way they talk with you and show you the importance of going to facility.

Response 3: I'm enjoying their services, they only hear of your pregnancy even before your pregnancy reaches two month ,they come visiting immediately to give advice on what you need to do stay healthy throughout your pregnancy period .

Response 4: of a truth they give us care. The give advice to prevent complication during pregnancy. Like sleeping in treated mosquitos' nets, that you take medication and injection as specified for you. And that you should attend ANC for your unborn and for your sake. The food you are expected to eat, the way to hold your child and the way you breast feed your child until he/she grow s up

Q8- What aspect of pregnancy and childbirth are the VHW not helping you with, that you would you like them to help you with?

Response 1: They are doing their best. God should bless them

Response 2: No problem, they are doing their best to enlighten us

Response 3: They are doing their best. I do not think there is any area

Probe: how can VHW reach more women like you?

All participants' response: They walk around, they are trying. They move from one place to the other

Response 1: They visit rugan Fulani, from one village to the other 3 to 4 times a week. Some places can only be access using motorcycles. They are suffering

Response 2: They do their best to reach more women, when they visit your area they want to have as many new registration of pregnant women for that month and you know they are many so that they can have some new registration to present at the end of the month. So that they have number of home and facility delivery to present

Q9-Do you understand the information the VHW convey to you?

All participants' response: we understand

Response 1: truly they teach us well. I understand their message. They emphasis hand washing, they talk about mosquito, danger signs in pregnancy this small thing and the need to go to facility but they stressed hand washing more.

Response 2: I understand their message is true. I enjoyed it there no problem. They talk about brushing, hand washing, keeping you home clean, washing your plates and dressing your beds and bathing at all times

Response 3: I understand their message very well. They talk about hand washing and keeping our surrounding clean, avoiding stagnant waters around our homes then going to facility and types of foods to be eaten.

Q 10-Do you understand the pictures in their flipcharts?

All participants' response: yes, I used to drop some with us

Response 1: They use to show women during ANC, pictures of women with various complications.

Response 2: they show us pictures of various classes of food and how these foods can be eaten. You don't need to disturb your husband to buy you meat, fish but you can make use of beans which can serve the purpose.

Q11-Do you feel free to ask VHW about any area you need clarification?

Response 1: I feel free to ask them because I know them, they are my friends.

Response 2: we grow up together, I have elder one's youngers one and my mates my friends as VHW so I'm free with them.

Q12- Has VHW visit to the community change your perception of facility delivery?

Response 1: it has changed my perception of facility delivery. They advised one appropriately.

Response 2: It has changed my perception then I only visit facility at the time of delivery only but now I attend ANC at early as 3 months of pregnancy at 2 month of pregnancy.

Q13-How do you feel about the fact that VHW are in your community?

Response 1: there is no problem. They escort one to facility during ANC and delivery. There is no problem having VHW in our locality.

Response 2: There is no problem. If you call VHW they respond on time and accompany you to the hospital

Q14-What do you like about VHW Program?

Response 1: I like the VHW because they are trying for us. I like their enthusiasm.

Response 2: I like the fact that they give drugs and one don't need to necessary visit facility at all time.

Response 3: like they say, I like their consistent visit and advice to go facility and their dressing

Response 4: like they all say I like the deliberate effort they put to reach to us always.

Q15-What do you not like about VHW program?

Response 1: I like everything about them.

Response 2: I like everything about the VHW that visit me at home. She teaches about taking care of your home, staying neat. This is what beautifies marriage. I like everything about her.

Response 3: There work is okay most especially the aspect of cleaning yourself and house.

Response 4: I like everything about their program. I'm concern for them walking daily under sun

Facilitator: This has brought us to the end of this discussion. Any question? Addition………

Response: I want their work to reach others. To expand their scope to reach others

Facilitator: thanks, you for time and answers. Our appreciation to your husbands for allowing you partake in this discussion.

End of Recording

Focus Group 5

Title: Focus Group Discussion with Mothers Who Delivered Their Babies in The Health Facility
Number of participants: 10

Main Discussion

Access to facility delivery

Q1: first, tell us a bit about how you travel to health facilities.

Response 1: I do trek to the health facility because it is not far from the hospital.

Response 2: I walk down to the hospital because it is close to my house.

Response 3: I use bike to come because it is a bit far, so I do pay transport.

Most of their houses are close to the facility.

Q2: How does your husband feel about you delivering your baby in the facility?

Response 1: my husband does allow me to come to the hospital, he allows me to come because he feels it safe and secure.

Response 2: my husband agrees to that he allows me to come to the hospital.

Response 3: my husband allows me deliver in the hospital because it makes him happy anytime I deliver in the hospital without complications.

Response 4: my husband does not have problem with that, he allows me because he thinks giving birth in the hospital is better than giving birth at home because you would be given proper treatment.

Response 5: he allows me because it is better, he feels it is better because if you give birth at home a complication might arise and they might not get the solution on time but if it is in the hospital, it will be treated immediately that is why he allows me give birth in the hospital.

Response 6: my husband agrees because you will be taken care of in the hospital.

Response 7: He agrees, he agrees because the hospital is a clean place and if the pregnancy will come with complications it will be taken care of before the I give birth, and also if I am bleeding there is a medicine that will be given to me but if I am to give birth at home, the baby might come out with complications that I don't even know because I gave birth to him in a dirty environment but in the hospital he would be cleaned and rubbed with cream and will be covered with a clean cloth.

Response 8: he agrees because he thinks is helpful, its helpful in such a way that the hospital is clean and when you give birth your baby will be taken care of, if it is at home there might be instance where water will enter the nose of the baby but if it is in the hospital, the workers won't let that happen.

Response 9: he feels happy when I deliver at the hospital because sometimes complications do occur when you deliver at home.

Response 10: he agrees because immediately you give birth in the hospital, your baby will be taken care of and the mother to know the condition of you two,

Q2: How does your mother in-law feel about you delivering your baby in the facility?

Response 1: she allows me deliver at the hospital because she feels it's safe because when you deliver, they do clean you up.

Response 2: the enjoys me giving birth at the hospital because she thinks it's good.

Response 3: she agrees because you and your baby will be checked properly in case of any complications.

Response 4: she agrees because you and your baby will be properly dress.

Response 5: she agrees because they check for any complications when you deliver in the hospital.

Response 6: she agrees it reduces complication like some diseases.

Response 7: my mother in-law agrees because she always tell her son that anytime I start labour I should be rushed to the hospital; she usually sees how they take care of me in the hospital.

Response 8: my mother is ok with it because the way they clean my body and my baby's own.

Response 9: she agrees because of the care I get from the hospital.

Response 10: she agrees because at home sometimes before a woman gives birth she might have convulsion or bleeding and they won't know the medicine to give her but if it's in the hospital, you will be treated before you give birth.

Q3: How do you feel when you came to the facility to deliver your baby?

Response 1: I do feel happy because of the care rendered to me at the facility, the take care of me immediately I come to the hospital till I give birth, the staff are nice and friendly, in the hospital you wear neat clothe unlike when you deliver at home.

Response 2: I feel good b because the hospital better than home delivery, they give attention immediately you come to the hospital till you put to bed, I don't think there is any problem with their work.

Response 3: I feel happy because they give me care and respect, they do not have any problem.

Response 4: I feel happy because I do not encounter any problem at the hospital, they staff take care of me immediately I come, I don't think they have any problem.

Response 5: I enjoy giving birth in the hospital, the staff handle us with care and respect. I want them to improve on ANC, because when women come they don't attend to us until the women become plenty and they will find it hard to attend to us on time, some women end up going home late at night and won't allow their wives go to the hospital again.

Response 6: I feel happy, the staff attends to us immediately, the respect us and talk to us politely.

Response 7: I feel happy because they attend to me immediately and they talk to me with respect, I don't think they have any problem.

Response 8: I feel happy because they take care of me immediately, I go to the hospital, they staff teat their patient with respect.

Response 9: I feel happy, they staff attend to me immediately I go, they handle me with care and respect, and their services are ok.

Q4: How is delivering in the facility different from delivering at home?

Response 1: better care is given at the hospital, like for instance if there is complications you would be given drugs but if it is at home no one will give you drugs.

Response 2:hospital delivery is better because there are instance when you will be feeling dizzy if it is in the hospital, you will be given drugs but if it at home nobody will give you drugs.

Response 3: better treatment would be given to you at the hospital

Response 4: there are medicine in the hospital which will be given to you anytime the need arise but there is no one at home for instance at home you will be bleeding , or drowsy but there is no drugs that will be given to you but in the hospital they can either give you drugs or injections.

Response 5: facility delivery is better because better care are given, when you give birth at the hospital, the person conducting the delivery usually wear hand gloves but at home the conduct it with bear hands and if the baby comes out, she wraps him in a clean cloth and lay him down in a clean place, and again if you come to the hospital, they do check whether it's time for delivery or not, if it's not time they ask you to go and come back but if it is at home, they do allow the child to fall on the ground before carrying him with dirty hands without gloves and lay him down in a dirty environment and if they notice it's not yet time for delivery they give the woman herbs to drink to induce the pregnancy.

Response 6: if you give birth in the hospital is better because even though you are to give birth at home if things get complicated you need to come to the hospital. If you give birth in the hospital you will be taken care of like they will clean you and your baby very well and if you have any problem, they will give you drug.

Response 7: if you give birth at home, they will give you herbs to be taking and sometimes it may lead to loss of life of either the mother or the baby, but if it is in the hospital they will check to know what is the problem.

Response 8: hospital is better they give better treatment and check if there are other problems

Q5: Do you have any special religious or special cultural requirements such as needing to be seen by a female healthcare provider?

Response 1: there is no religion that said it must be a man or woman that will conduct the delivery and those that collect the delivery here are mostly women, I would like a woman to conduct the delivery, if it's a woman if I have any problem I can tell her without hesitation, or shame, they woman treats me well without any problem

Response 2: I would prefer a woman not because of religion or culture but because a woman is my sister I can tell her anything but if it's a man, I will be shy to talk to him, a woman knows everything but if I should go to the hospital without meeting a female I would allow the male conduct the delivery.

Response 3: I prefer a woman not because of religion or culture but because if I have any problem I won't hide it to her but if it's a man I would be ashamed, normally it's a woman that do conduct delivery for me and she handles me with care and respect.
Response 4: I prefer a woman because she would be more concern, she do look after me anytime I come for delivery, I don't have any problem with her.
Response 5: I prefer a woman not because of religion or culture but because she will look after me properly.
Response 6: I prefer a woman because I can tell her anything disturbing me, she do welcome properly.

Q6: What do you think can be done to facilitate you to deliver in the health facility?

Response 1: advice women and tell them the importance of giving birth in the hospital, advice like, if you go for ANC they will tell you what is wrong with you and the position of your child if he is not laying down well or if the baby is not moving if you stay at home you won't know all this and also if you don't have enough blood in your body you will also be told, if she understand clearly what you mean she will start going to the hospital and even tell others.
Response 2: advice should be given to the women to enable them go to the health facility, the ones that will prevent them from contacting diseases, the road is not a problem it is good the hospital services are ok, money can be a problem for those who are because they need to pay for transportation and other necessities when they come to the hospital like drugs and other things so at times if they remember this they feel discouraged to come to the health facility.

Probe: What do you think can be done to facilitate other women to deliver in the health facility?

Response 1: the TBA should visit them frequently and tell the danger in not going to the hospital, the husbands too need to be told on the importance of giving birth in the health facility, some women will love to go but their husbands won't allow them because they feel they will spend much money.
Response 2: the men should be included in meetings even if it's not going to be together with the women so that they can be told the importance of going to the facility because most of the women are being denied going to the facility by their husband.
Response 3: let the TBA meet the men and discuss with them, they should give them advice and some vital information on the importance of going to the health facility, if this is done anytime his wife is pregnant and in labor he will remember the advice and allow her go to the hospital, advices like whenever your wife is pregnant and you notice her bleeding or water coming out of her private part take her to the hospital so that they will know what the problem is.
Response 4: the women should be given advice to go to the hospital because there are times where a woman needs blood in her body but she would not know because she didn't go to the hospital, some women are willing to go to the hospital but lack of money is what is stopping them.

Response 5: their husbands should be told so that they can allow them come to the hospital, some complications like headache, stomach pain in sufficient blood in the body can be noticed when you come to the hospital, if you come to the hospital they check you and prescribe drugs for to go and buy but some wont buy because of lack of money, .
Response 6: they should invite their husbands and tell to allow their wives go to the hospital that is if they have money.

Q7: How do you feel about the VHWs?
Response 1: we feel happy about them and they do tell us the importance of ANC and what will happen if you don't go, they also show us some danger signs that we need to take note of like swelling of the body, bleeding, lack of blood, abdominal pain, sever back pain and many more, any time we notice this signs we should rush to the hospital.
Response 2: there is no problem with their work because they do visit us at home, and they do tell us to keep our surrounding clean, we should go to the hospital always so that we can know the position of our child if he is not laying well those at the facility will ask us to go to the big hospital so that they can repair it for us.
Response 3: I feel happy about their work, they are been sent to visit us at home and tell us the importance of going to the hospital
Response 4: I am happy because they do visit us at home and show us some pictures and some danger signs in case of complications like headache, stomach pain, fever etc if we notice this signs we should go to the hospital and explain to the doctors so that they can give us drugs.
Response 5: I am enjoying their visit because they do tell us interesting things we need to know, things like if we ate not feeling fine we should go to the hospital so that they can give us drugs and they do tell us what to eat that can add blood to our body like , egg orange and vegetables.
Response 6: I feel happy because they do tell us to go to the hospital so that we can know our wellbeing and that of our child.
Response 7: I feel happy about their work because they do advise us to for ANC and to deliver in the hospital.
Response 8: I feel happy about their visitation because they do tell us to for ANC.

Q8: What aspect of pregnancy and childbirth are the VHW not helping you with?
Response: Their work is particularly good; they teach everything pertaining childbirth and pregnancy.

Probe: How can VHW reach more women like you?
Response 1: They should visit them more frequently and teach them about health facility, they should show them the pictures in their book and explain to them.
Response 2: they should add more effort to their work because if you visit a woman once and she refuses to attend to you, when you go subsequently she will attend to you, the VHW should tell her to go for ANC she should also tell her the benefit and

dangers of ANC, some women if you go the first time she would ignore you because she doesn't know the importance of it so she should go ba ck again to remind her.
Response 3: they should always remind the women about the importance of going to the health facility.
Response 4: if she tells them once and they didn't hear, she should go back again.
Response 5: they are trying because they do enter every woman's house.

Q9: Do you feel you understand the information the VHW conveys to you when she visits you?
Response: I understand every information they tell me, and I use to feel free to ask her any question, there is no difference with what we are being told at the hospital.
We do understand everything the teach us.

Q10: Do you feel you understand the flip chart the VHW uses for health education.
Response: I do understand I the flip chart, and it is not different with the one in the hospital.

Q11: Do you feel free to ask VHW questions about the information she delivers to you?
Response 1: I do ask her question if I don't understand like for instance the case of ANC I do ask her about it, I ask her about everything and don't feel shy or somehow to ask her any Probe.
Response 2:I understand all the pictures she shows me, she do ask me question and I also ask her question if I don't understand, I do ask her like for instance if I give birth and I start bleeding what will I do? She does respond well to my question and there is no Probe I cannot ask her.
Response 3: I do understand everything, and I do ask question like for instance if I am having body pain should I go to the hospital or I should buy drugs in the chemist? And she will tell me what to do, I do not think there is anything I can't ask her.
Response 4: I do understand the flip chart and I do ask her question if I do not understand like may be my hand is swollen what will I do? She will answer me well, there is no question I cannot ask her.

Q12: Has VHW visits to the community changed your perception of facility deliveries?
Response 1: it has changed from the aspect of ANC, giving birth in hospital and also keeping our environment clean.
Response 2: it has help in keeping our environment clean.

Q13: How do you feel about the fact that the VHWs are members of your community?
Response 1: its better because if anything happens, we can call her at any time since we are in the same community, I don't think it's a problem because she's from our community.
Response 2: she is closer to us anytime we feel something we can call her immediately.

Q14: What do you like about the VHW program?

Response 1: I like the work because they tell us the benefit of going to the hospital.

Response 2: they do tell us to clean our surrounding and drink clean water.

Response 3: their work has created awareness a lot before we don't give birth in the hospital but now, we do go, and we are happy about that.

Response 4: they do tell us what to eat that will build our body and add blood to our body, and to clean our surrounding and drink clean water.

Response 5: before there are a lot of things, we do not know but now we know because they do visit us at home and tell us wat do

Response 6: I enjoy their work because they teach us a lot of things like ANC

Response 7: they do tell us what to do that will make us strong and healthy during pregnancy

Response 8: they do tell us to clean our surrounding and eat clean food.

Q15: What do you not like about the VHW program?

All participants' response: We like everything about the program.

Focus Group 6

TITLE: FOCUS GROUP DISCUSSION WITH MOTHERS WHO DELIVERED THEIR BABIES AT HOME
DURATION OF RECORDING: 1HR 15MINS
NUMBER OF PARTICIPANTS: 10

Q1. **Can you tell us why you do not come to the facility for delivery?**

Response 1: we always come for antenatal but when it's time for delivery we have our TBA who attends to us at home.
Response 2: I didn't deliver at the facility because there was no one to bring me to the facility when I was in labour.
Response 3: I went into labor at the middle of the night before dawn I had delivered m y baby.
Response 4: when I was in labour there was no body at the facility
Response 5: the facility workers were on strike when I was in labor that was why they called the TBA to take my delivery.
Response 6: I had my two kids during Christmas when all the staffs were on break.

Q2. **How does your husband feel about you delivering your baby in the facility?**

Response 1: my husband feels happy it because it has to do with my health but he is not always around, so whenever am in labor I call on the TBA who live close to my house instead of going to the facility for delivery.
Response 2: my husband feels happy because at the facility they will check my health and that of the baby but when am about to come to the facility for delivery the baby comes out early than expected so they will just call on the TBA to attend to me.
Response 3: my husband support facility delivery because of the extra care we get when we deliver and when there is an emergency they take proper care of the situation but Allah always bring my delivery at home, that is why all my delivery is at home I have never birth my babies at the facility.
Response 4: my husband support facility delivery but I deliver my babies at home because the facility is mostly on strike when am in labor.
Response 5 : my husband feels good about facility delivery because even when am feeling headache he will always tell me to go to the facility because of good health, but I usually have a short labor there by delivering my babies after a short labor.
Response 6: my husband feels good about it but my first two deliveries falls during Christmas so when he brought me to the facility there was no one to attend to me, when I was about to deliver my third child the labor was short so I delivered at home.

Q2. How does your mother in law feel about you delivering your baby in the facility?

Response 1: she like it because of the health of the mother and child but most times before they call her, I already deliver my baby.
Response 2: my mother in law support my delivery at the facility in fact she is the one who will tell her son to hurry and get a bike that will take me to the facility but when we get to the facility its either they are on Christmas break or strike or the facility worker has gone home for wedding.
Response 3: she supports me because whenever we deliver at home, she gets angry with us.

Response 4: she supports me because of the health of the baby and the mother
Response 5: my mother in law is late
Response 6: she likes it but when am in labor and they call her before she gets to my place, I already deliver my baby.
Response 7: before God she likes it but before we talk about taking me to the facility, I already deliver my baby.
Response 8: I do not know my mother in law
Response 9: I do not know my mother in law too
Response 10: my mother in law is not staying in the same community with me usually she just gets to hear that I have delivered.

Q3. How is delivering at the facility different from delivering at home?

All participants' response: there is a difference
Response 1: At the facility, if a woman in labor has convulsion or shortage of blood they will take care of the situation by transfusing blood and other things but if the delivery was to be at home the TBA won't know what to do about it thereby causing havoc to the mother or child.
Response 2: at the facility they will make sure the baby and the mother are in good health but when you deliver at home they will need to bring the baby to the facility again to be checked if he is fine or not, then when there is any problem with the mother too drugs will be given to her but when the delivery takes place at home the TBA will refer them to the facility for proper care.
Response 3: at the facility if during labor a woman is short of water or blood, she will be given all she needed but when the delivery takes place at home no TBA can do that.
Response 4: at the facility the baby and mother are well taken care of but at home if any emergency arise, they will still need to be brought back to the facility for proper care.
Response 5: there is a difference, concerning the stomach pain that happens after delivery which cause dizziness which call throw one away but if the delivery is at the facility the will render help to such a woman on time.
Response 6: at the facility they will give drugs if there is a problem but there are no drugs to give to the woman after delivery if there is a problem.
Response 7: if you deliver safely at the delivery and there is a problem, they will tell you and take care of you.
Response 8: before delivery the water that comes out of the woman body sometimes these water smells if the delivery is at the facility they will help give what will stop the smell but when the delivery is at home there is no drugs to stop such smell.
Response 9: at the facility they will always check the health of the baby.

Q4. Do you have any special religious or cultural requirements such as needing to be seen only by a female healthcare provider?

All participants' response: no
Response 1: I prefer a female worker to attend to me because it's only my fellow woman who knows the secret of women.
Response 2: for me, any one on duty can take my delivery.
Response 3: anybody can take my delivery as far as God gives me good health.
Response 4: I prefer a female worker to take my delivery

Response 5: for me I will prefer a female worker because she is a woman like me but in a situation where there is no female worker on duty then I can allow a male worker to take my delivery.

Q5. What do you think can be done to facilitate you to deliver in the health facility?

Response 1: laughs… for me i don't know

Response 2: to make women come to the facility, when they come for antenatal the facility workers should be friendly to pregnant women and give us drugs all these will want the women come to the facility to meet with the friendly facility workers, the should also give the pregnant woman gifts. There is no problem with the roads to the facility; the facility does not have any problem as well it is a sure place to get care when pregnant. Also, money is not a barrier to the facility

Response 3: they should explain to the women the difference when they deliver at the facility and at home

Response 4: the should help us reduce the money they collect at the hospital after delivery, they should help us also by giving us drugs for free these will make women want to deliver at the facility and also the facility workers should be friendly and accommodating.

Response 5: because of the constant 'lauleyi' (illness) pregnant women do have if drugs are been given to them to stop it when they get home and tell the other women about the drugs these will draw their attention to the facility. Money is not the problem because money is meant to be spent.

Response 6: when the women who deliver at the facility get home and tell the other women how they care for them during delivery these will make the other women to want to deliver at the facility.

Response 7: when we come for antenatal to avoid boredom or tiredness the facility workers can tell us to clap or sing with these activities, we can draw the attention of other women to the facility for delivery.

Q6. How do you feel about the VHWS?

Response 1: the create awareness among women especially those who don't like going to the facility because of this we like their work.

Response 2: their work is top notch when we are pregnant they check on us, give us drugs and if there is need to visit the facility they tell us.

Response 3: they are really trying; they tell us the importance of neatness when pregnant and why we need to visit the facility regularly.

Response 4: they show us a lot of things they tell us the food to eat while pregnant and they show us ways to improve our health and that of the baby.

Response 5: there work is correct, they show us things from the leaflet they give us.

Response: we are enjoying their work because some pregnancy comes with complications, sometimes you won't be able to eat but with the help of the leaflet they gave us which shows the food a pregnant woman should eat when we show our husbands they get us those foods we need during pregnancy.

Response 6: they are trying because they always tell us the need to visit the facility for antenatal care.

Q7. What aspect of pregnancy and childbirth are the VHW not helping you with, that you would like them to help you with?

Response 1: there is none

Response 2: they should continue to put more effort on the job they are doing.

Probe: How can VHW reach more women like you?

Response 1: when they enter into a house and there is a pregnant woman who don't like going to the facility , if they tell them that there will be gift for them when the go to the facility that will make them curious thereby drawing there attention to the facility.

Response 2: we can point out the houses of pregnant women we know so they can enter and tell them the importance of antenatal and facility.

Response 3: more women can be reached if they are told drugs at the facility are free.

Response 4: the help you need is from us the women since we are the ones that go out for occasions such as wedding and naming ceremony, we can recognize pregnant women and point them out for VHW.

Response 5: when gifts are given to women who deliver at the facility and their friends who deliver at home saw it and ask where they get it from, if they tell them at the facility when next they want to deliver they will go to the facility so they can get that gift too.

Response 6: more women can be reach when awareness is created at occasions such as naming ceremony

Q8. Do you feel you understand the information the VHW conveys to you when she visits you?

All participants' response: We understand every information the VHW conveys to us and there is no difference between from the information we get from the provider in the health facility.

Q9. Do you feel you understand the flip chart the VHW uses for health education?

All participants' response: Yes, we see this kind of chart in the health facility

Q10. Do you feel free to ask VHW Probes about the information she delivers to you?

Response 1: I ask her the reason for neatness, and I ask her about health in general, there is no Probe I can't ask her.

Response 2: I don't ask them any Probes except if I don't understand what they said earlier and there are no Probes I can't ask them.

Response 3: I only ask them to explain again if I don't understand them.

Response 4: as a pregnant woman you might have different problems, I sometimes asked them what to do when am not feeling fine and they will tell me to always go to the facility to complain. There is no Probe I can't ask them because they are woman just like me

Response 5: you know their different Probes in different batches as a pregnant woman we ask them all these Probes.

Q11. Has VHW visits to the community changed your perception of facility delivery?

Response 1: when your fellow woman comes to deliver at the facility and you see the way she was cared for during and after delivery and you also see the gift they gave her, when it's time to deliver you also will want to deliver at the facility so you can get such care.

Response 2: Eh…. It is good when they tell the necessary information and the information is of great use which shows their explanation is of great importance

Response 3: yes, because the process of delivery before is different from what we have now, before there are so many diseases but now there is a great reduction in diseases because of the help women got at the facility.

Response 4: yes, because we don't even come for ANC before but we now come for ANC because they told us the importance.
Response 5: their visit is enjoyable because when they enter our houses they will tell us different things and when we do as they say we stand to gain a lot, they try their best going from house to house and we enjoy the information they keep passing.
Response 6: their work is very good, they pass good information on women that don't go the facility, before these women don't come to the facility but they now comes to the facility due to the awareness created by these VHWs.

Q13. How do you feel about the fact that the VHWs are members of your community?

All participants' response: We feel good because we are used to them and they pass good information around, we don't have any problem with the fact that they are from our community.
Response: it is a good thing they are from our community because they tell us all we need to know and in case of emergency they are closer because they stay in the community and they can be called upon at any time of the day we need their services.

Q14. What do you like about the VHW program?

All participants' response: We are happy about the VHW work because they create awareness among pregnant women, they give important information and tell us things we don't know before,
Response 1: we enjoy all these, and we always feel like they should not leave when they visit us at our various homes.
Response 2: I enjoy all their work; I also enjoy the fact that they go from house to house to inform pregnant women. There is nothing about their work that I don't like.

Q15. What do you not like about the VHW program?

All participants' response: All their explanation is on point
Response: there is nothing we do not like about the program, and we want to say a big thank you to them we pray that Allah bless them all.

End of Recording
