# Supplementary material for: Assessing the acceptability of village health workers’ roles in improving maternal health care in Gombe State, Nigeria a qualitative exploration from women beneficiaries
Source: PLoS One. 2020 Oct 22;15(10):e0240798. doi: 10.1371/journal.pone.0240798 (PMC7580965; doi:10.1371/journal.pone.0240798)
Supplement: S5 File — (PDF) [file pone.0240798.s005.pdf]

No. Item	Guide questions/description	Reported on page #
Domain 1: Research team and reflexivity		
1. Inter viewer/facilitator	Which author/s conducted the interview?	9
2. Credentials	What were the researcher's credentials?	9
3. Occupation	What was their occupation at the time of the study?	9
4. Gender	Was the researcher male or female?	9
5. Experience and training	What experience or training did the researcher have?	9
6. Relationship with participants established	Was a relationship established prior to study commencement?	9
7. Participant knowledge of the interviewer	What did the participants know about the researcher?	10
8. Interviewer characteristics	What characteristics were reported about the inter viewer/facilitator?	9
Domain 2: study design		
9. Methodological orientation and Theory	What methodological orientation was stated to underpin the study?	7
10. Sampling	How were participants selected?	8
11. Method of approach	How were participants approached?	8
12. Sample size	How many participants were in the study?	9
13. Non-participation	How many people refused to participate or dropped out? Reasons?	9
14. Setting of data collection	Where was the data collected?	7 & 8
15. Presence of nonparticipants	Was anyone else present besides the participants and researchers?	11
16. Description of sample	What are the important characteristics of the sample?	12,13 & 14
17. Interview guide	Were questions, prompts, guides provided by the authors?	9 & 10
18. Repeat interviews	Were repeat interviews carried out?	11
19. Audio/visual recording	Did the research use audio or visual recording to collect the data?	11
20. Field notes	Were field notes made during and/or 10 after the interview?	11

21. Duration	What was the duration of the interviews	11
22. Data saturation	Was data saturation discussed?	11
23. Transcripts returned	Were transcripts returned to participants for comment and/or correction?	11
Domain 3: analysis and findings		
24. Number of data coders	How many data coders coded the data?	11 & 12
25. Description of the coding tree	Did authors provide a description of the coding tree?	11 & 12
26. Derivation of themes	Were themes identified in advance or derived from the data?	11
27. Software	What software, if applicable, was used to manage the data?	11
28. Participant checking	Did participants provide feedback on the findings?	11
29. Quotations presented	Were participant quotations presented to illustrate the themes/findings? Was each quotation identified?	15-22
30. Data and findings consistent	Was there consistency between the data presented and the findings?	15-22
31. Clarity of major themes	Were major themes clearly presented in the findings?	15-22
32. Clarity of minor themes	Is there a description of diverse cases or discussion of minor themes?	15-22
